# Aqueous Electrochemical and pH Studies of Redox-Active Guanidino Functionalized Aromatics for CO_2_ Capture

**DOI:** 10.1021/acsorginorgau.3c00066

**Published:** 2024-03-22

**Authors:** Clarabella J. Li, Joseph W. Ziller, Jeffrey M. Barlow, Jenny Y. Yang

**Affiliations:** Department of Chemistry, University of California, Irvine, 1102 Natural Sciences II, Irvine, California 92697-2025, United States

**Keywords:** electrochemistry, pH Swing, redox-active guanidine, CO_2_ capture, electroswing

## Abstract

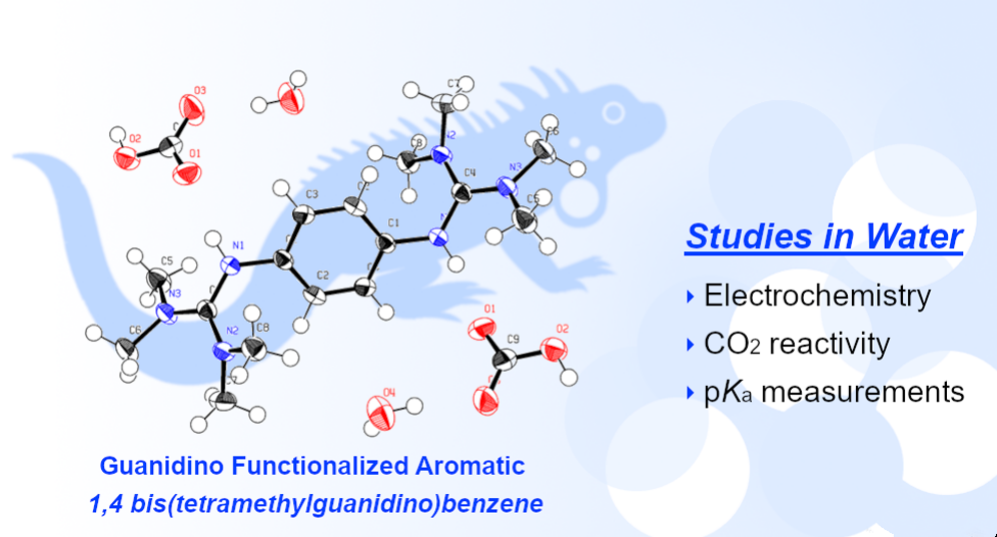

Escalating levels of carbon dioxide (CO_2_) in the atmosphere have motivated interest in CO_2_ capture and concentration from dilute streams. A guanidino-functionalized aromatic 1,4-bis(tetramethylguanidino)benzene (1,4-btmgb) was evaluated both as a redox-active sorbent and as a pH swing mediator for electrochemical CO_2_ capture and concentration. Spectroscopic and crystallographic studies demonstrate that 1,4-btmgb reacts with CO_2_ in water to form 1,4-btmgbH_2_(HCO_3_^–^)_2_. The product suggests that 1,4-btmgb could be used in an aqueous redox pH swing cycle for the capture and concentration of CO_2_. The synthesis and characterization of the mono- and diprotonated forms (1,4-btmgbH^+^ and 1,4-btmgbH_2_^2+^) and their p*K*_a_ values were measured to be 13.5 and 11.0 in water, respectively. Electrochemical pH swing experiments indicate the formation of an intermediate radical species and other degradation pathways, which ultimately inhibited fully reversible redox-induced pH cycling.

Each year, about 40 gigatons of carbon dioxide is emitted from human activity, with roughly 30% of these emissions arising from fossil fuel power plants.^1,2^ Flue gas from fossil fuel power plants typically contains 8–10% of CO_2_. The current state-of-the-art method for the capture of CO_2_ from flue gas uses an aqueous alkanolamine solution to capture CO_2_ at ambient temperature and pressure. The release of CO_2_ is achieved by heating the sorbent (383 K for monoethanolamine).^3,4^ However, these thermal-swing methods can only achieve 5–40% of the maximum theoretical energy efficiency.^1,5^

Electrochemical methods can achieve greater theoretical energy efficiencies.^4,6^ One common motif for ambient electrochemical CO_2_ capture and concentration uses redox active molecules that either (1) directly bind to and release CO_2_ or (2) modify the pH of aqueous solutions (Scheme 1A).^7^ In the first approach, redox carriers bind CO_2_ in their electron-rich reduced form (Scheme 1A, red pathway). Subsequent electrochemical oxidation decreases its electron density, lowering its CO_2_ binding affinity and releasing concentrated CO_2_. The redox carrier can then be regenerated for CO_2_ capture via electrochemical reduction.^8^ Another approach uses redox chemistry to change the pH of an aqueous solution for CO_2_ capture and release (Scheme 1A, blue pathway). These pH swing cycles capitalize on pH-dependent CO_2_ equilibria in aqueous solutions. At high pH values, hydroxide reacts with CO_2_ to form bicarbonate and carbonate, enabling capture from dilute streams. At low pH values, the equilibrium is reversed and CO_2_ is released. Redox-active molecules whose proton affinity (p*K*_a_) significantly varies depending on their oxidation state are used to induce these electrochemically driven pH swings.^9^

**Scheme 1:**
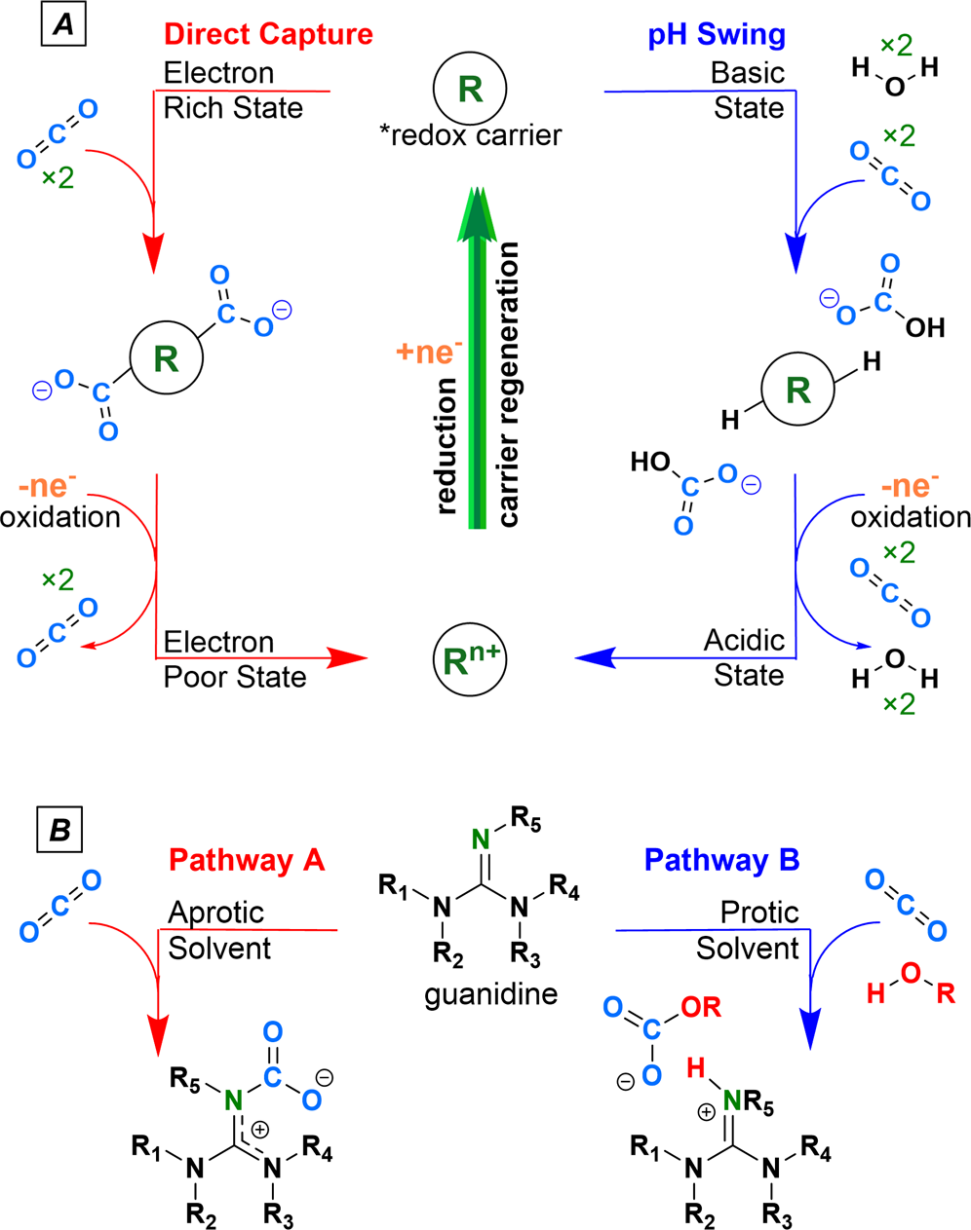
Two Modes of CO_2_Capture; A (Top): Two Modes of CO_2_ Capture by a Redox-Active Molecule (R) via Direct Capture or pH Swing Methods; B (Bottom): Two Modes of CO_2_ Capture by Guanidines

Guanidines are a family of neutral organic Bro̷nsted superbases (p*K*_a_ values of 12 or greater in water) with attractive CO_2_ capture properties.^10^ They bind CO_2_ through two different mechanisms, depending on the solvent. These mechanisms parallel the two capture routes shown in Scheme 1A.^8^ In aprotic solvents, tetramethyl guanidine (TMG) binds directly to CO_2_ to form a zwitterionic carbamate salt (Scheme 1B, pathway A).^11^ In protic solvents such as alcohols or water, sufficiently Bro̷nsted basic guanidine deprotonates the alcohol or water, which then binds CO_2_ to form an alkyl carbonate or carbonate ion pair with the protonated TMG (Scheme 1B, pathway B).^12^

Because of their reactivity with CO_2_, guanidine derivatives have been studied for their use in thermal capture and release cycles. Heldebrandt and co-workers developed a new class of CO_2_-binding organic liquids composed of alcohol solvents and TMG derivatives.^12^ Direct air capture (DAC) of CO_2_ by a family of bis-iminoguanidine has been studied by Custelcean and co-workers.^13−15^ The resulting bis-iminoguanidine carbamate salts can be heated to regenerate the sorbent (80–120 °C).^14,15^

The lack of reversible redox properties in the previously studied guanidine compounds limited their use as sorbents in thermal regeneration cycles. However, Himmel and co-workers recently synthesized TMG-substituted functionalized aromatics, or guanidino functionalized aromatics (GFAs), with reversible redox properties.^16^ These redox-active superbases have not been assessed for electrochemical CO_2_ capture and release. A variety of GFAs can be obtained through straightforward synthetic routes. Among these, 1,4-bis(tetramethylguanidino)benzene (1,4-btmgb) was selected for testing due to its mild redox potential and stability in air. In this study, we explored the use of 1,4-btmgb in organic and aqueous solutions to assess its viability as a redox carrier and pH swing mediator for electrochemical CO_2_ capture.

## Results and Discussion

### Synthesis of GFAs

**Scheme 2:**
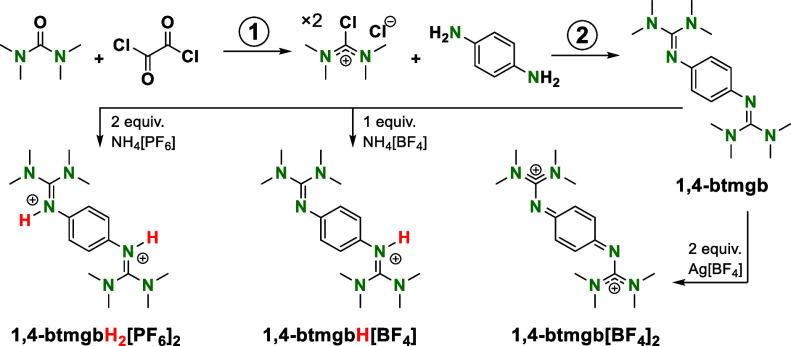
Synthesis of 1,4-btmgb, 1,4-btmgb^2+^, and 1,4-btmgbH_2_^2+^

1,4-btmgb and the doubly oxidized form 1,4-btmgb[BF_4_]_2_ were synthesized as described by Himmel and co-workers (Scheme 2).^16^ In addition, the previously unreported conjugate acids [1,4-btmgbH_2_]^2+^ with PF_6_^–^ and Cl^–^ counteranions and monoprotonated [1,4-btmgbH]^+^ with BF_4_^–^ or PF_6_^–^ counteranions were also synthesized. These protonated compounds were characterized via ^1^H and ^13^C{^1^H} NMR spectroscopy (Figures S1–S3) and X-ray crystallography (Figures S4–S5).

### p*K*_a_ and Electrochemical Properties in Acetonitrile

**Figure 1:**
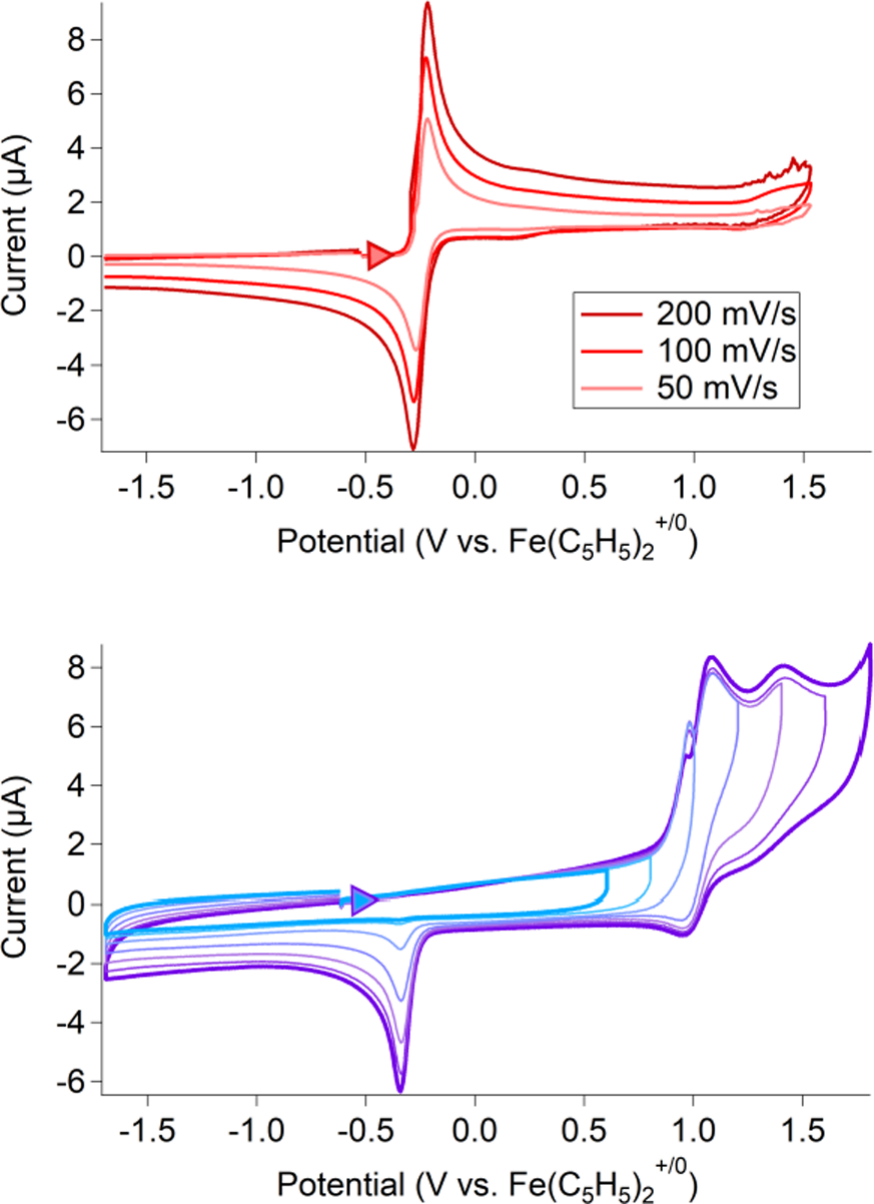
Top: 1,4-btmgb (1 mM) in MeCN with TBAPF_6_ as the supporting electrolyte (100 mM) at varied scan rates. Bottom: varied scan window of 1,4-btmgbH_2_[PF_6_]_2_ (scan rate = 500 mV/s) in MeCN, with 100 mM TBAPF_6_ as the supporting electrolyte and a glassy carbon counter electrode. These and the following CVs were carried out at room temperature and are reported following the IUPAC convention.

The p*K*_a_ values for the doubly protonated 1,4-btmgb, [1,4-btmgbH_2_]^2+^ were previously reported in acetonitrile (MeCN) as 19.3 and 22.1, respectively.^17^ In MeCN, the cyclic voltammogram (CV) of the fully deprotonated 1,4-btmgb has a reversible two-electron oxidation with an *E*_1/2_ at −0.25 V vs ferrocenium/ferrocene (Fc^+/0^) (Δ*E* = 0.030 V) (Figure 1, top). This redox behavior is consistent with the previous reports of 1,4-btmgb in other organic solvents referenced to ferrocene, which described a reversible two-electron reduction at −0.18 V in DCM and −0.26 V in THF.^16^

The CV of the doubly protonated species, [1,4-btmgbH_2_]^2+^, is shown in Figure 1, bottom (and Figure S7). This species has irreversible oxidation events at the more anodic potentials of 1.12 and 1.39 V vs Fc^+/0^. After oxidation, the subsequent reduction at −0.33 V is similar to the potential for the reduction of [1,4-btmgb]^2+^ seen in the CV of deprotonated [1,4-btmgb]^2+^/1,4-btmgb (Figure 1, top). Oxidation of [1,4-btmgbH_2_]^2+^ by two electrons is expected to result in an increase in acidity. We believe that [1,4-btmgbH_2_]^4+^ (formed upon the oxidation of [1,4-btmgbH_2_]^2+^) is deprotonated, despite the lack of an added base, to form the more stable 1,4-btmgb^2+^. The CV trace for the monoprotonated species (Figure S8) maintains a one-electron redox couple centered at −0.24 V, a lone reduction event at 0.39 V, and oxidation peaks at 1.19 and 1.30 V vs Fc^+/0^.

### Reactivity with CO_2_

**Figure 2:**
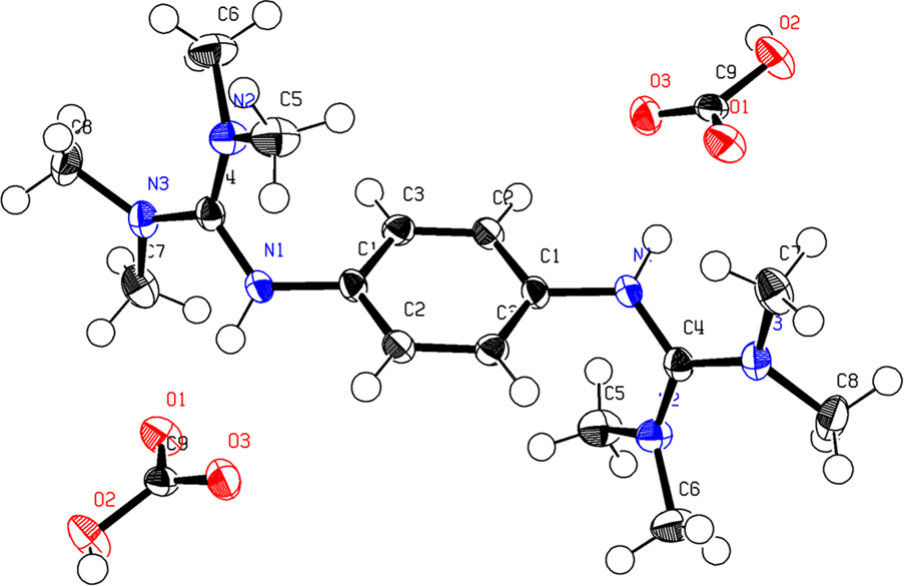
X-ray crystal structure of 1,4-btmgbH_2_(HCO_3_^–^)_2_. Thermal ellipsoids are shown at 80% probability. Refinement data can be found in SI Tables S1–S7.

The CO_2_ capture capability of 1,4-btmgb was assessed in MeCN, with CO_2_ being added to solutions of 1,4-btmgb under different conditions. Sparging CO_2_ into a 1 mM solution of 1,4-btmgb in MeCN with trace water resulted in the formation of a white precipitate. Single crystals of this product, suitable for analysis by X-ray crystallography, were grown from a solution of concentrated 1,4-btmgb dissolved in MeCN under a CO_2_ headspace. The analysis reveals the formation of protonated 1,4-btmgb with bicarbonate anions (1,4-btmgbH_2_(HCO_3_^–^)_2_) (Figure 2). Other attempts to recrystallize 1,4-btmgb with CO_2_ in MeCN resulted in a similar structure, but with two water molecules to give 1,4 btmgbH_2_(HCO_3_^–^)_2_·(H_2_O)_2_ (Figure S6).

The formation of a carbonate salt was also present in pure water, supported by ^1^H NMR analysis. The ^1^H NMR spectra of 1,4-btmgb in D_2_O has signals at δ 6.85 and 2.82 ppm which shift to 7.11 and 2.97 ppm, respectively, after sparging with CO_2_ for 5 min (Figures S9 and S10). After CO_2_ exposure, these signals are identical to those found for doubly protonated 1,4-btmgbH_2_[PF_6_]_2_ in D_2_O (Figures S11 and S12). The ^13^C{^1^H} NMR of 1,4-btmgb in D_2_O with added CO_2_ is the same as that of 1,4-btmgbH_2_[PF_6_]_2_ with two additional resonances observed at δ 124.8 and δ 160.3 ppm which correspond to dissolved free CO_2_ in solution and bicarbonate (HCO_3_^–^), respectively.^18,19^

### p*K*_a_ and Electrochemical Properties in Protic Solvents

**Figure 3:**
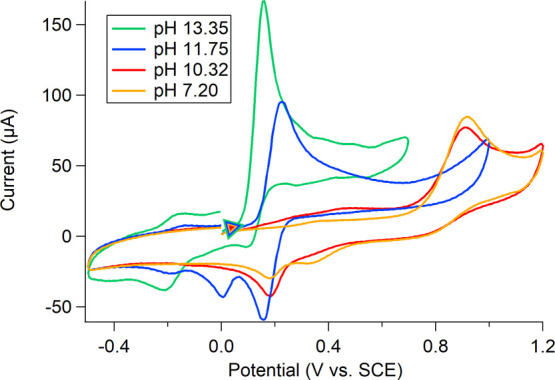
CVs of 1,4-btmgb in 100 mM KCl in H_2_O at various pH values.

**Figure 4:**
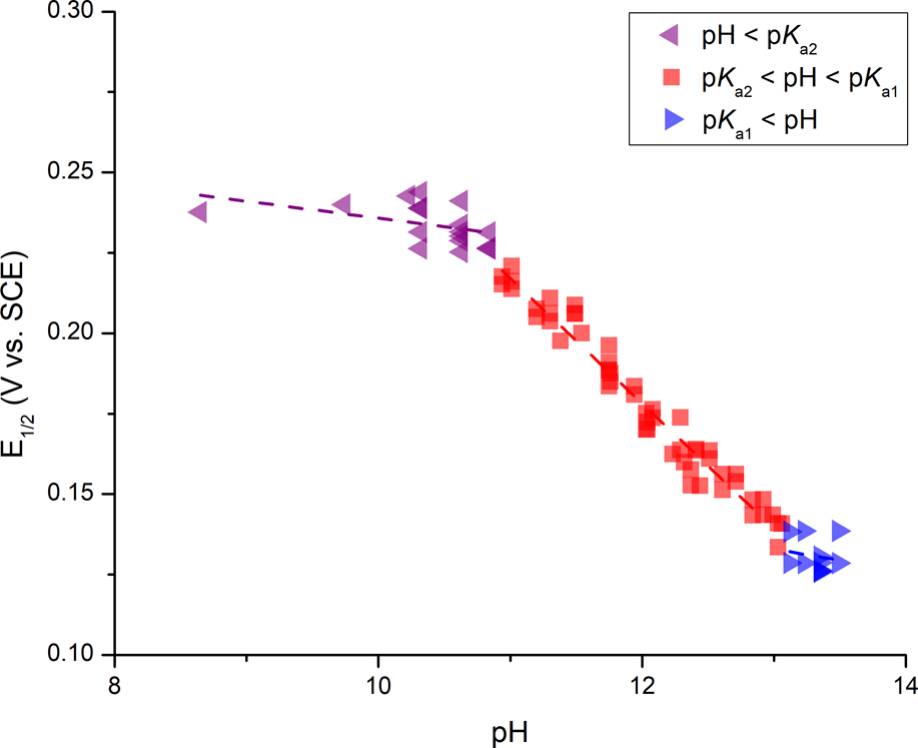
pH vs *E*_1/2_ (V vs SCE) from points collected from titrating 1 M acetic acid and 1 M NaOH into 1,4-btmgb in aqueous KCl (conc.) solution.

Since even trace water in aprotic solvents results in the formation of the protonated base and bicarbonate, we postulate that 1,4-btmgb is a better candidate for an aqueous electrochemical pH swing cycle than direct capture. To assess the suitability of 1,4-btmgb for an aqueous electrochemical pH swing, we first sought to determine the p*K*_a_ values for [1,4-btmgbH_2_]^2+^ in water. Compared to the CVs obtained on 1,4-btmgb at various stages of protonation in MeCN (Figure 1), the CVs of 1,4-btmgb in water varied with pH (Figures 3 and S15–S19), reflecting different protonation states. The relationship between the electrochemical potential and the solution pH with 1,4-btmgb was used to determine its p*K*_a_ values.^20−22^ Cyclic voltammograms of 1,4-btmgb were acquired at pH values between 2.5 and 13.8. From these experiments, a partial Pourbaix diagram (Figure 4) was constructed by plotting the pH measured to the observed *E*_1/2_ (or *E*_pa_ and *E*_pc_, Figures S20–S22).^23^ The reduction potential remained relatively constant below the pH value of 11. The oxidation potential shifted cathodically as the pH increased from 11 to 13.5 (Figures 4 and S20–S22). The slope of the shift in *E*_1/2_ versus pH was observed to be −40.4 mV/pH, indicating a two-electron, one-proton process.^23^ Above pH 13.5, there were minimal changes in the redox potential. Further supporting this, with differential-pulse voltammetry (DPV) experiments from pH 9.5 to 14.1, the exact breaking points in the pH-dependent and pH-independent regions allowed for the determination of p*K*_a_ values (Figures S23 and S24). Collectively, these data indicate a p*K*_a1_ value of approximately 13.5 ([1,4-btmgbH]^+^) and a p*K*_a2_ value of 11.0 ([1,4-btmgbH_2_]^2+^).

The redox features of 1,4-btmgb are less reversible at both higher and lower pH values. Between pH 11.3–11.9 there were two additional reduction peaks negative of the reversible redox event at *E*_1/2_ = 0.2 V vs saturated calomel electrode (SCE) (pH = 11.75) (Figure 3, blue trace). The reduction event at 0.0 V is attributed to the reduction of [1,4-btmgbH]^3+^, from the oxidized [1,4 btmgbH]^1+^ that is expected to form at these pH values. The more cathodic reduction event at −0.18 V vs SCE increases in current at high pH (11.3 and higher, Figure 3, green trace) but was not observed when 1,4-btmgb was probed electrochemically in other nonaqueous protic solvents (methanol and ethanol, Figures S25–S26). We attribute this reduction to an interaction with water upon oxidation, which is supported by electrolytic studies (vide infra).

### Controlled Potential Electrolysis and Supporting Experiments

Electrochemically induced pH changes with 1,4-btmgb and [1,4-btmgbH_2_][Cl_2_] were attempted through controlled potential oxidative electrolysis in a divided H-cell. Two mM aqueous solutions of 1,4-btmgb were prepared. 1,4-btmgb is sufficiently basic to deprotonate water. As a result, the initial pH of the solution was 11.71 (±0.06). Controlled potential electrolysis was carried out at 0.5 V vs SCE to fully oxidize the [1,4-btmgbH]^1+^ to [1,4-btmgbH]^3+^ and [1,4-btmgb]^2+^. The final pH was observed to be 9.50(±0.21).

**Figure 5:**
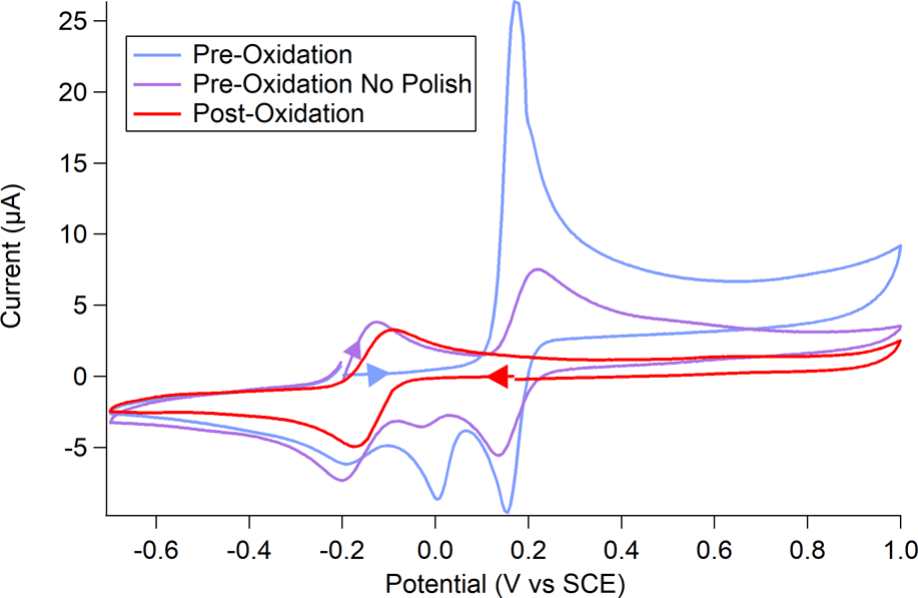
Two mM **1,4-btmgb** in 200 mM KCl and water, preoxidation scan rates 500 mV/s and postoxidation scan rate 250 mV/s.

An aqueous 2 mM solution of protonated [1,4-btmgbH_2_][Cl_2_] was also oxidized. The initial pH was 9.88 and then decreased to 8.72. Upon subsequent reduction, the pH of the solution increased to 9.38 (Table 1). While both solutions produced a change in pH from electrolysis, the magnitudes were smaller than expected and the solutions could not be restored to the original pH after reduction. The solutions also turned deep red during oxidation. The color faded in intensity over time but did not completely disappear. The CVs of the oxidized 1,4-btmgb solution are shown in Figure 5 (red trace). Compared to the trace of the solution before oxidation, a new reductive event appears at −0.18 V vs SCE. This feature and the color change indicate the formation of new species after controlled potential oxidative electrolysis.

**Table 1:** pH Values Observed before and after Electrolysis of 2 mM Solutions of 1,4-btmgb and 1,4-btmgbH_2_^2+^^a^

compound	initial pH	post oxidation	post reduction
1,4-btmgb	11.71 (±0.06)	9.50 (±0.21)	10.50
1,4-btmgbH_2_^2+^	9.88	8.72	9.38

a Electrolysis performed stirred in a divided H-cell, with a carbon fabric working electrode and SCE reference electrode on the working side and a glassy carbon electrode on the counter side. Further details of the electrolysis in the methods.

**Scheme 3:**
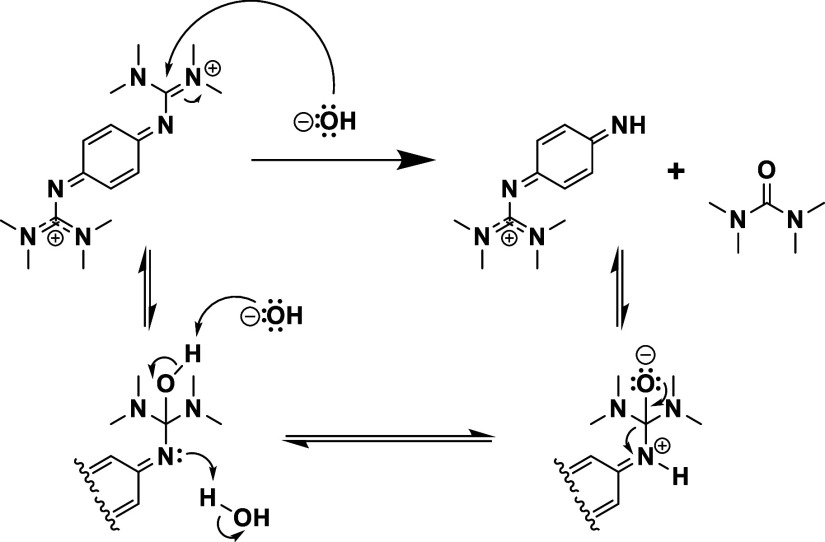
Alkaline Hydrolysis of a TMG Group on Oxidized 1,4 btmgb

The redox event at −0.18 V vs SCE we attribute to an aniline species that is the product of the decomposition of 1,4-btmgb via the loss of a TMG group via base-catalyzed hydrolysis (Scheme 3). This reduction feature is present only in CVs of 1,4-btmgb in water among the protic solvents surveyed, indicating it is related to the [1,4-btmgb^2+^] generated in situ interacting with water/hydroxide. Prior studies have likened the redox behavior of the TMG substituted aniline to that of 1,4-bis(dimethylamino)-benzene where oxidation of this compound results in the Wurster cation and undergoes further decomposition in the solution mixture due to the N–H moiety.^24^ There is literature precedence for this route of decomposition to form tetramethyl urea (Scheme 3).^25−27^^1^H NMR experiments were also performed on chemically oxidized [1,4-btmgb^2+^] in deuterated acetonitrile in the presence of water, showing the decomposition of the parent species 30 min after the addition of water. Over the course of 5 h, additional resonances at around 2.89 ppm and in the aromatic region (6.74–6.76 ppm) increase in intensity (Figures S27 and S28). The resonance at 2.89 ppm is assigned to the methyl proton peaks of tetramethyl urea, and the resonances between 6.74–6.76 ppm are attributed to the aniline species formed post hydrolysis (tetramethylguanidino-dimethyl-*p*-phenylenediamine has aromatic resonances at 6.49–6.69 ppm while *p*-phenylenediamine has a resonance at 6.46 ppm in CD_3_CN).^24^ Altogether, the new resonance peaks in the aromatic region indicate decomposition in the presence of water. Monitoring the reduced species 1,4-btmgb in D_2_O at high and low pH via ^1^H NMR spectroscopy does not show evidence of decomposition over the span of days, indicating it is the oxidized species that is sensitive to deleterious reactions with water.

Further evidence for decomposition with hydroxide upon oxidation is observed in the scan-rate dependent CVs. A new reductive feature appears after oxidation of [1,4-btmgbH]^3+^ and the [1,4-btmgb]^2+^ species (Figure S13). This reduction event at −0.18 V vs SCE increases with scan rates 250 mV/s and above and slower for solutions over pH 11.5 (Figures S13–S16). This reductive feature does not appear if the solution is not first oxidized.

**Scheme 4:**
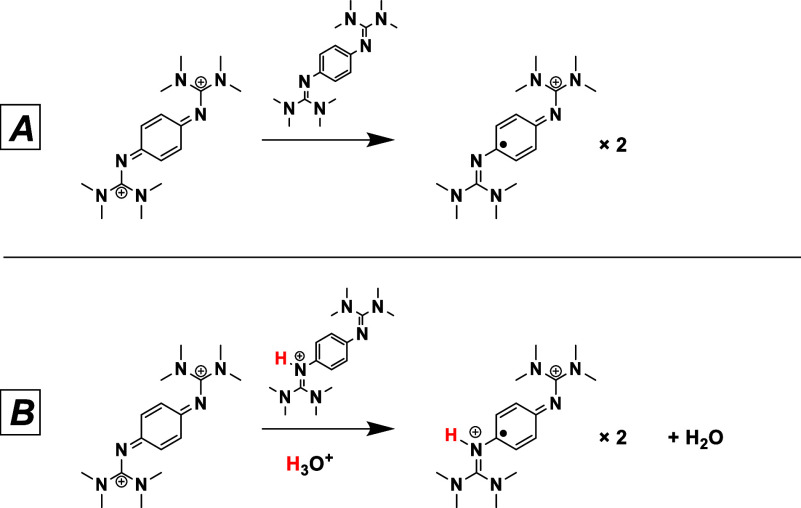
Various Proposed Comproportionation Pathways for [1,4-btmgb]^2+^ (A) Unprotonated and (B) between a Reduced Monoprotonated Species [1,4-btmgbH]^+^

In addition to the hydrolysis decomposition pathway in Scheme 3, we attribute the color change observed during electrolysis to a second decomposition pathway that results in radical formation. Prior studies in acetonitrile conditions have shown that the fully oxidized [1,4-btmgb]^2+^ can comproportionate with 1,4-btmgb to form [1,4-btmgb^·^]^1+^ in solution (Scheme 4).^28^ Water is also known to stabilize this radical formation.^29^

To verify the presence of a radical in the solution, electron paramagnetic resonance (EPR) spectra were acquired by using aliquots of the oxidized 2 mM 1,4-btmgb. An EPR signal at *g*_⊥_ = 2.0066 was present for the solution after controlled potential electrolysis at 0.5 V for 10 min, indicating the formation of an *S* = 1/2 organic radical species (Figure S29).

**Figure 6:**
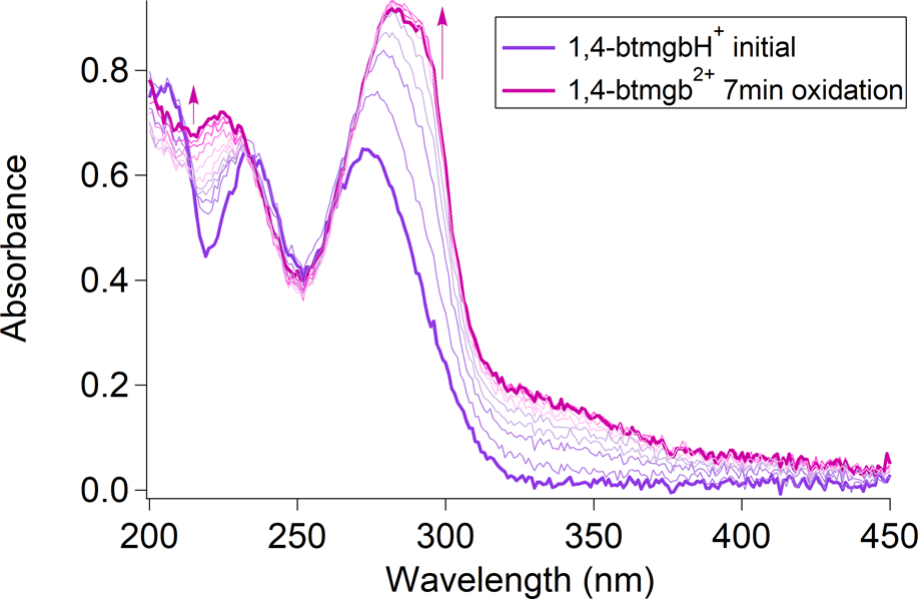
UV–visible spectroelectrochemical data of 0.1 mM 1,4-btmgb. Oxidation at 500 mV vs Ag^+^/Ag electrode in unbuffered water with KCl (100 mM) was performed at pH 11.91. Under these conditions, it is expected to protonate once to give 1,4-btmgbH^+^. Purple trace is initial 1,4-btmgbH^+^ and pink trace is oxidized 1,4-btmgb^2+^ at 250 mV vs SCE.

Ultraviolet–visible (UV–vis) spectroelectrochemistry (SEC) was used to obtain absorption spectra of the generated oxidized species [1,4-btmgb]^2+^ (Figure 6) through controlled potential electrolysis of a 0.1 mM solution of 1,4-btmgb with 100 mM KCl in water at 0.25 V vs SCE. 1,4-btmgbH^+^ has absorbances of 235 and 272 nm. Oxidation leads to two new absorbances at 224 and 291 nm, which have higher absorbance.

The postelectrolysis solution obtained after oxidation in an H-cell with stirring leads to different absorption spectra than what were observed in the UV–vis SEC experiments (Figure S30). The absorbances that correspond to the oxidized species were not present. We believe that convection increases the rate of comproportionation. After 1 day, the absorbance at 272 nm also decreases in intensity, indicating decomposition.

The solubility of 1,4-btmgb was measured in water in the absence and presence of CO_2_. Unlike MeCN, it was found that the addition of CO_2_ does not diminish the solubility of 1,4-btmgb in water or lead to precipitation, even in saturated solutions. The determined solubility of 1,4-btmgb in water is 97 g/L (0.32 M) (Figures S31 and S32).

Collectively, these decomposition pathways result in smaller than expected pH changes in the controlled potential electrolysis experiments (Table 1). Additionally, the side reactions hinder the regeneration of 1,4-btmgb and the overall reversibility of an electrochemical cycle.

## Conclusions

A guanidine-functionalized aromatic (GFA) compound, 1,4-btmgb, was examined as a redox-active candidate for CO_2_ capture. Reactivity with CO_2_ in the presence of trace water forms protonated guanidine and carbonate. In addition, the stability of 1,4-btmgb and the preliminary redox behavior in water indicate that it could potentially mediate a pH swing cycle. The aqueous p*K*_a_ values of [1,4-btmgbH_2_]^2+^ were measured to be 11.0 and 13.5. However, controlled potential electrolysis indicates more complicated reactivity in water with hydrolysis and radical formation decomposition pathways. The use of GFAs in electrochemical CO_2_ capture and concentration will require addressing these decomposition pathways.

## Experimental Section

### General Information

Synthetic work was carried out in either ambient air or under a dinitrogen (N_2_) atmosphere in a glovebox where noted. All solvents and reagents were purchased from commercial vendors and used without further purification unless otherwise noted. Deuterated acetonitrile used for NMR characterization were purchased from Cambridge Isotope Laboratories, Inc., and were degassed via free–pump–thaw and stored over activated 3 Å molecular sieves. Tetrabutylammonium hexafluorophosphate (TBAPF_6_) was purified via recrystallization from ethanol and dried under a heated (80 °C) vacuum (1 × 10^–3^ Torr) and stored in a glovebox. Experiments using carbon dioxide (CO_2_) atmospheres were performed using ultrahigh purity (99.999%) CO_2_ which was additionally passed through a purification column to eliminate residual H_2_O, O_2_, CO, halocarbons, and sulfur compounds.

### Electrochemical Methods

Electrochemical experiments were carried out under an atmosphere of N_2_ or CO_2_, where indicated. Solutions for electrochemical measurements were recorded in acetonitrile or water using 100 mM TBAPF_6_ (or TMAPF_6_) or 100 mM KCl as the supporting electrolytes, respectively. Cyclic voltammetry (CV) was performed with a Pine instrument WaveDriver 10 potentiostat. When specified, ferrocene (Fc) was used as an internal standard and potentials are referenced to the ferrocenium/ferrocene (Fc^+/0^) couple. In water, a saturated calomel electrode (SCE) was used as the internal reference electrode. Between CV scan, the working electrode was polished in a figure eight pattern with an alumina slurry (0.05 um) on a microcloth (2.875 in, PSA backed). Controlled potential electrolysis experiments were performed stirred in a custom H-cell with the working and the counter cells divided by a porous glass frit. In the working compartment, carbon fabric was used as the working electrode and SCE as the reference. A glassy carbon rod was used as the counter electrode on the counter side cell. UV–visible spectroelectrochemistry (UV–vis SEC) was performed using a UV–vis SEC kit from Pine Instruments with a Pt working/counter electrode and Ag wire as a pseudo reference electrode.

### Physical Methods

UV–vis absorption spectra were recorded by using a 1 cm quartz cuvette with an Agilent Cary 60 UV–vis spectrophotometer. CO_2_ monitoring was performed on a CM-0111 gas sensor kit CM-0111 by CO_2_Meter with the software GasLab. A Bruker EMX spectrometer equipped with an ER041XG microwave bridge was used to collect the X-band EPR spectra. Fourier Transform Infrared (FTIR) spectra were collected by using a Thermo Scientific Nicolet iS5 FTIR Spectrometer with iD5 diamond ATR. ^1^H, ^13^C{^1^H}, and ^19^F{^1^H} NMR spectra were recorded at 500 MHz on Bruker or 600 MHz Varian instruments. ^1^H NMR spectra chemical shifts were reported as δ values in ppm relative to the residual solvent: D_2_O (4.79 ppm), MeOD (3.31 ppm), and CD_3_CN (1.94 ppm). pH values were collected with a Thermo Scientific Orion Star A216 pH/RDO/DO meter. The instrument was calibrated with Thermo Scientific buffers at pH 4.01, 7.00, and 10.01 each time before use.

X-ray diffraction studies were conducted at the UCI Department of Chemistry X-ray Crystallography Facility on a Bruker SMART APEX II or Bruker X8 Prospector diffractometer. Crystals were mounted in a cryoloop and transferred to a Bruker X8 Prospector (SMART APEX II, Mo Kα, λ = 0.71073 Å) or Bruker X8 Prospector (Cu Kα, λ = 1.5406 Å) diffractometer system. The APEX3^1^ (APEX2^1^) program package was used to determine the unit-cell parameters and for data collection (2 s/frame scan time). The raw frame data was processed using SAINT^2^ and SADABS^3^ to yield the reflection data file. Subsequent calculations were carried out by using the SHELXTL^4^ program package. There were no systematic absences nor any diffraction symmetry other than the Friedel condition. The structure was solved by direct methods and refined on *F*^2^ by full-matrix least-squares techniques. The analytical scattering factors^5^ for neutral atoms were used throughout the analysis. Hydrogen atoms were located from a difference-Fourier map and refined (*x*, *y*, *z*, and *U*_iso_). The molecule was located about an inversion center.

#### 1,4-Bis(tetramethylguanidino)benzene

Synthesized following previously described prep by Himmel and co-workers.^16^^1^H NMR (600 MHz, CD_3_OD) δ = 6.63 (s, 4H) 2.72 (br, 24H) ppm. ^1^H NMR (600 MHz, D_2_O, unbuffered) δ = 6.85 (s, 4H), 2.82 (s, 24H) ppm. ^13^C{^1^H} (151 MHz, D_2_O, unbuffered) δ = 161.2, 139.9, 122.5, 39.19 ppm.

#### 1,4-btmgb[BF_4_]_2_

Synthesized following previously described prep by Himmel and co-workers, but using Ag[BF_4_] instead of Ag[PF_6_].^16^ Use in water decomposition experiments in CD_3_CN as seen in Figures S27 and S28.

#### 1,4-btmgbH_2_[PF_6_]_2_

In a glovebox, a mixture of 1,4-btmgb (0.018 g, 0.06 mmol) and [NH_4_][PF_6_] (0.016 g, 0.1 mmol) in dry MeCN (20 mL) was stirred for 45 min while heated at 55 °C. The solvent was removed under reduced pressure, and the resulting solid was redissolved in MeCN, yielding a clear yellow solution. Activated carbon was added, and the solution was left to stir for 15 min before being filtered through glass fiber. Removal of the solvent yielded a white powder that, when recrystallized from Et_2_O, formed clear crystals. Yield: 14 mg (41%). ^1^H NMR (600 MHz, CD_3_CN) δ = 7.93 (s, 2H), 7.04 (s, 4H), 2.92 (s, 24H) ppm. (600 MHz, D_2_O) δ = 7.11 (s, 4H), 2.97 (s, 24H) ^13^C{1H} NMR (151 MHz, CD_3_CN) δ = 159.3, 135.6, 123.3, 40.8 ppm. 13C{1H} (151 MHz, D_2_O) δ = 158.6, 134.9, 122.3, 39.6 ppm. UV/vis (CH_3_CN): λ_max_ (ε in L·(mol cm)^−1^) = 277 (4.0 × 104), 231 (2.2 × 104) nm. FTIR (ATR)/cm^–1^ ν = 3400 (w), 3328 (w), 2926 (w), 1627 (m), 1563 (w), 1520 (w), 1466 (w), 1420 (m), 1316 (w), 1230 (w), 1165 (w), 1067 (w), 1041 (w), 917 (w), 822 (s), 741 (m), 557 (m). MS (ESI-MS) *m*/*z* calcd C_16_H_29_N_6_^+^ [M-H-2(PF_6_)]^+^ 305.25, found *m*/*z* 305.25. (Low-res MS) found *m*/*z* [M-PF_6_]^+^ 451.3, [M-H-_2_(PF_6_)]^+^ 305.3, and [M/2]^2+^ 153.2.

#### 1,4-btmgbH_2_[CO_3_H]_2_

Preparation varied, but for the general isolation of the compound, the procedure for characterization and experiments is as follows. A solution of 1,4-btmgb (10 mg) in MeCN (5 mL) was sparged with CO_2_ for 3 min for CO_2_ saturation at room temperature. While the solution was still actively sparged by CO_2_, deionized water (0.1 mL) was added to the solution and a cloudy precipitate was observed to form. The solution was dried under a vacuum, yielding a chalky white power. Yield: quantitative. Crystals suitable for X-ray diffraction were grown from dissolved 1,4-btmgb in MeCN and drops of water, under a headspace of CO_2_. ^1^H NMR (600 MHz, CD_3_OD) δ = 6.94 (s, 4H), 2.90 (s, 24H) ppm. ^1^H NMR (600 MHz, D_2_O) δ = 7.11 (s, 4H), 2.97 (s, 24H) ppm. ^13^C{^1^H} NMR (151 MHz, D_2_O) δ: 160.3, 158.6, 134.9, 124.8, 122.3, 39.6 ppm. FTIR (ATR)/cm^–1^ v = 3380 (w), 3010 (w), 2917 (w), 2876 (w), 2802 (w), 2161 (w), 1564 (s), 1461 (s), 1437 (m), 1372 (s), 1268 (m), 1230 (m), 1204 (m), 1132 (s), 1097 (m), 1062 (w), 1016 (s), 922 (w), 841 (s), 751 (m), 685 (m), 638 (w), 578 (w). MS (ESI-MS) *m*/*z* calcd C_16_H_29_N_6_^+^ [M-H-2(CO_3_H)]^+^ 305.25, found *m*/*z* 305.25. (low-res MS) found *m*/*z* [M-H-2(CO_3_H)]^+^ 305.3, [M/2]^2+^ 153.2.

#### 1,4-btmgbH[BF_4_]

In a glovebox, a mixture of 1,4-btmgb (0.021 g, 0.07 mmol) and [NHBu_3_][BF_4_] (0.019 g, 0.07 mmol) in dry MeCN (20 mL) was stirred for 1 h at 55 °C. The solvent was removed under reduced pressure, and the resulting beige solid was recrystallized from MeCN and Et_2_O. Yield: 10 mg (50%). ^1^H NMR (600 MHz, CD_3_CN) δ = 7.79 (s, 1H), 6.73 (s, 4H), and 2.76 (s, 24H) ppm. ^13^C{^1^H} NMR (151 MHz, CD_3_CN) δ = 159.1, 123.1, 122.3, 115.0, 39.4 ppm. ^19^F{^1^H} NMR (565 MHz, CD_3_CN) δ = 151.8 ppm. FTIR (ATR)/cm^–1^*v* = 3382 (w), 3340 (w), 2932 (w), 2887 (w), 2361 (w), 2163 (w), 1630 (m), 1551 (m), 1493 (m), 1383 (m), 1318 (w), 1231 (w), 1205 (w), 1144 (w), 1049 (s), 1019 (s), 914 (w), 855 (m), 843 (m), 764 (w), 616 (w), 579 (w). MS (ESI-MS) *m*/*z* calcd C_16_H_29_N_6_^+^ [M-BF_4_]^+^ 305.25, found *m*/*z* 305.25.

## Data Availability

The data underlying this study are available in the published article and its Supporting Information.

## Supporting Information Available

In the order they are referenced in the main text, the SI contains characterization data (^1^H, ^13^C NMR, UV–vis, EPR, and FT-IR spectra), crystallographic structures and refinement data, additional CVs, and details of the DPV and p*K*_a_ determination experiments (PDF)

## Abbreviations

btmgb: bis(tetramethylguanidino)benzene
CO_2_: carbon dioxide
CV: cyclic voltammetry
DAC: direct air capture
DPV: differential-pulse voltammetry
EPR: electron paramagnetic resonance
GFA: guanidino functionalized aromatics
SCE: saturated calomel electrode
TMG: tetramethyl guanidine.
