# Supplementary material for: Aqueous Electrochemical and pH Studies of Redox-Active Guanidino Functionalized Aromatics for CO2 Capture
Source: ACS Org Inorg Au. 2024 Mar 22;4(4):387–94. doi: 10.1021/acsorginorgau.3c00066 (PMC11311035; doi:10.1021/acsorginorgau.3c00066)
Supplement: Supplementary file 1 — gg3c00066_si_001.pdf [file gg3c00066_si_001.pdf]

Supporting Information

Aqueous Electrochemical and pH Studies of Redox-Active Guanidino Functionalized Aromatics for CO_2 Capture

Clarabella J. Li, Joseph W. Ziller, Jeffrey M. Barlow, and Jenny Y. Yang*

Department of Chemistry, University of California Irvine
1102 Natural Sciences II, Irvine, CA 92697-2025

Index ***Page***

^1H and ^{13}C NMR spectra …… **S2-3**

Crystallographic structures…… **S3-4**

Cyclic Voltammetry of 1,4-btmgb$H_2[PF_6]_2$ and 1,4-btmgbH$[PF_6]$…… **S5**

NMR experiments monitoring CO_2 addition…… **S6-7**

Cyclic Voltammetry of 1,4-btmgb in water at various solution pH…… **S8-11**

pH vs. E_{pc}, pH vs. E_{pa}, and DPV data…… **S11-14**

Cyclic Voltammetry of 1,4-btmgb in MeOH and EtOH…… **S15**

NMR experiments of $[1,4\text{-btmgb}]^{2+}$ in CD_3CN with water…… **S16-17**

EPR data…… **S18**

UV-Vis spectra of 1,4-btmgb…… **S18**

Solubility Data…… **S19-20**

FT-IR spectra…… **S20-22**

Crystallography Refinement Data…… **S23**

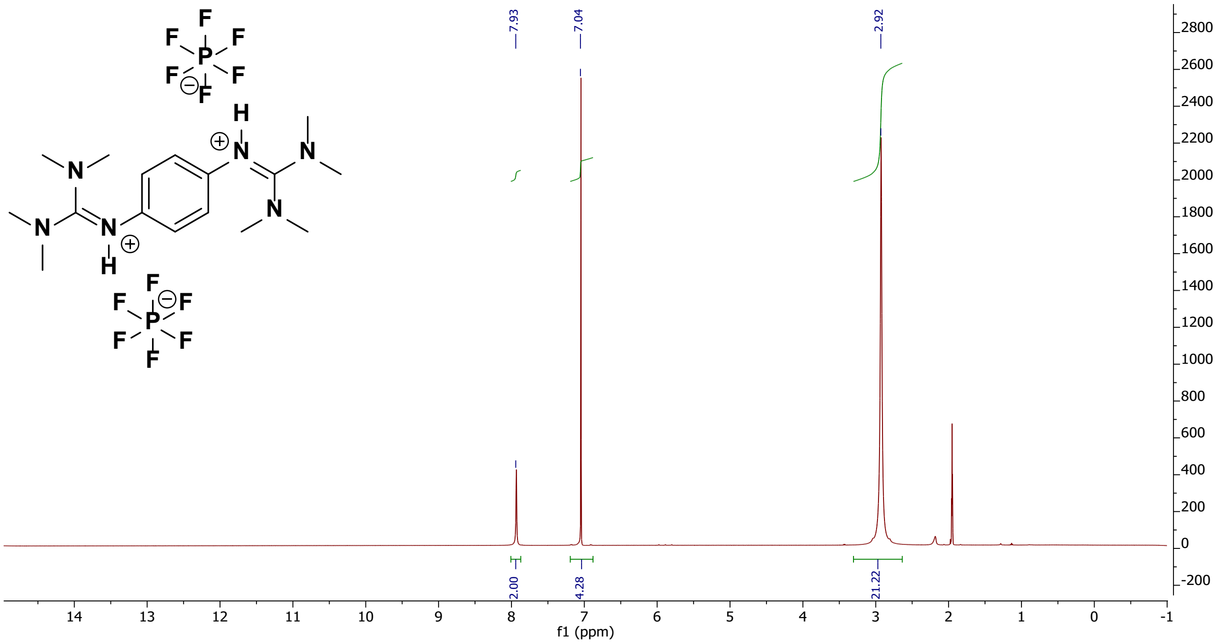

Figure S1. ^{1}H NMR of **1,4 btmgbH$_2$[PF$_6$]$_2$** in CD_3CN, 600 MHz, 298 K.

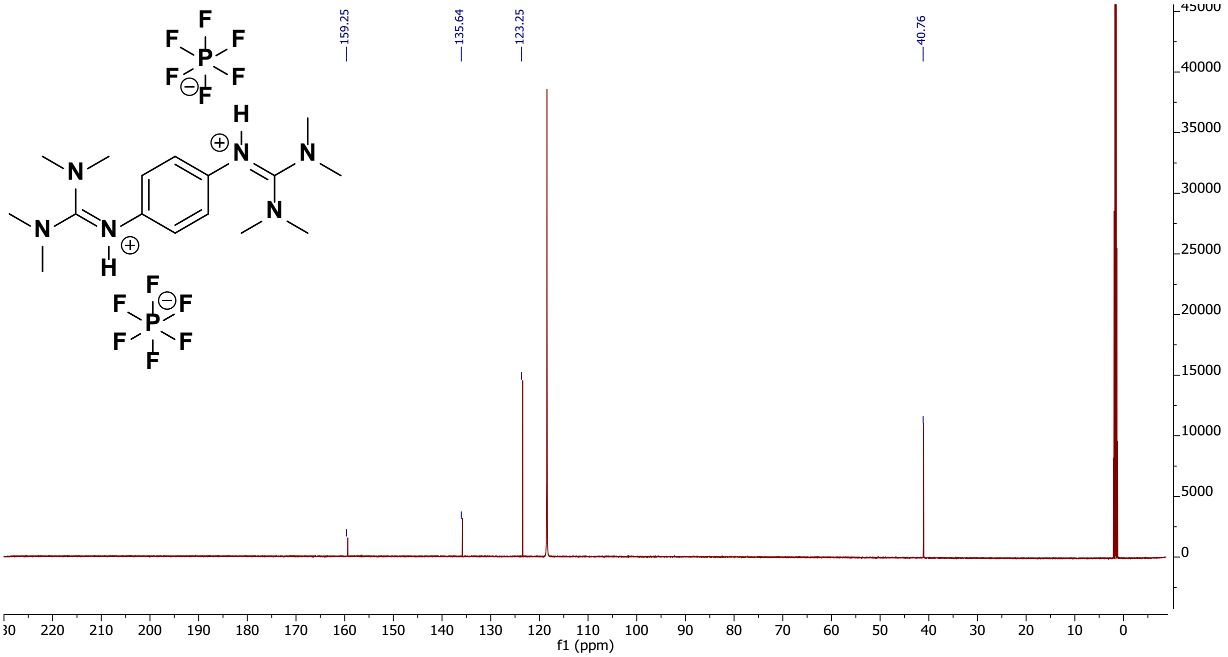

Figure S2. $^{13}C\{^{1}H\}$ NMR of **1,4 btmgbH$_2$[PF$_6$]$_2$** in CD_3CN, 600 MHz, 298 K.

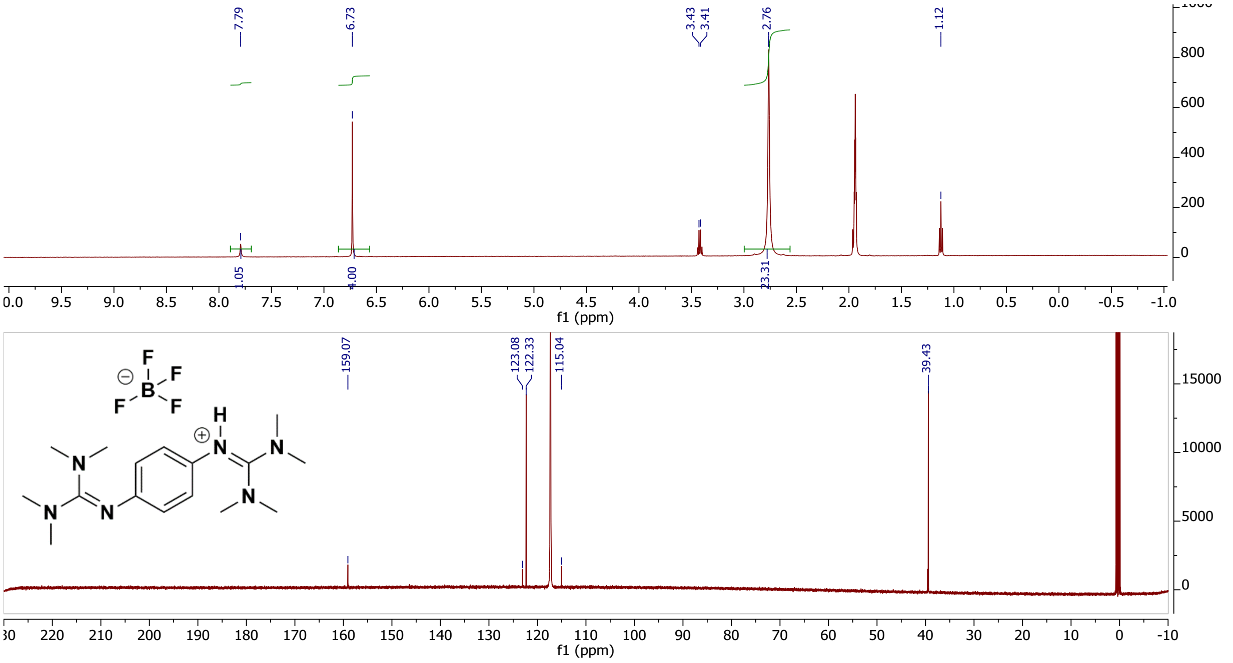

Figure S3. [1]H (500 Hz) and ^{13}C{^{1}H} (151 MHz) NMR of **1,4 btmgbH[BF$_4$]** in CD_3CN, 298 K.

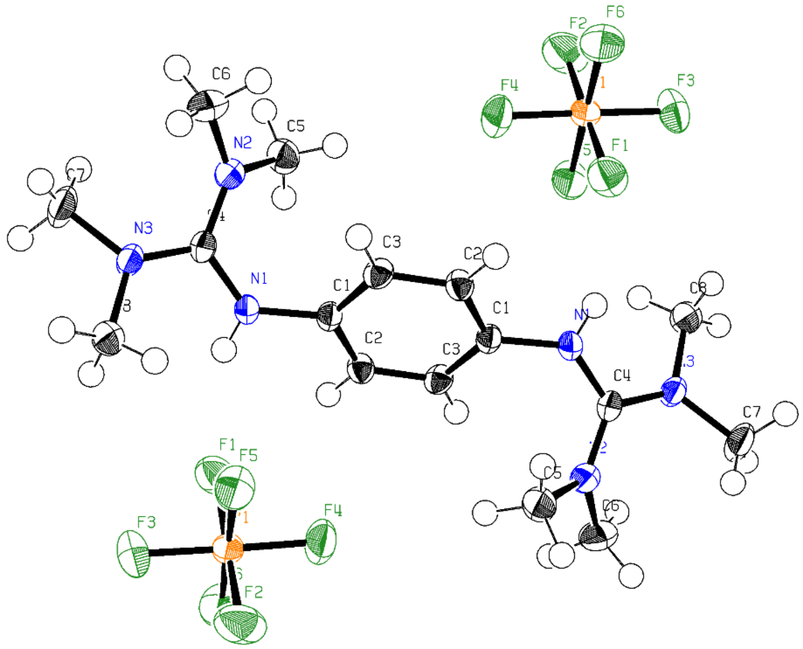

Figure S4. ORTEP structure of **1,4 btmgbH$_2$[PF$_6$]$_2$**. Thermal ellipsoids are shown at 80% probability. See refinement data tables **S8-S13** starting on page **S27.**

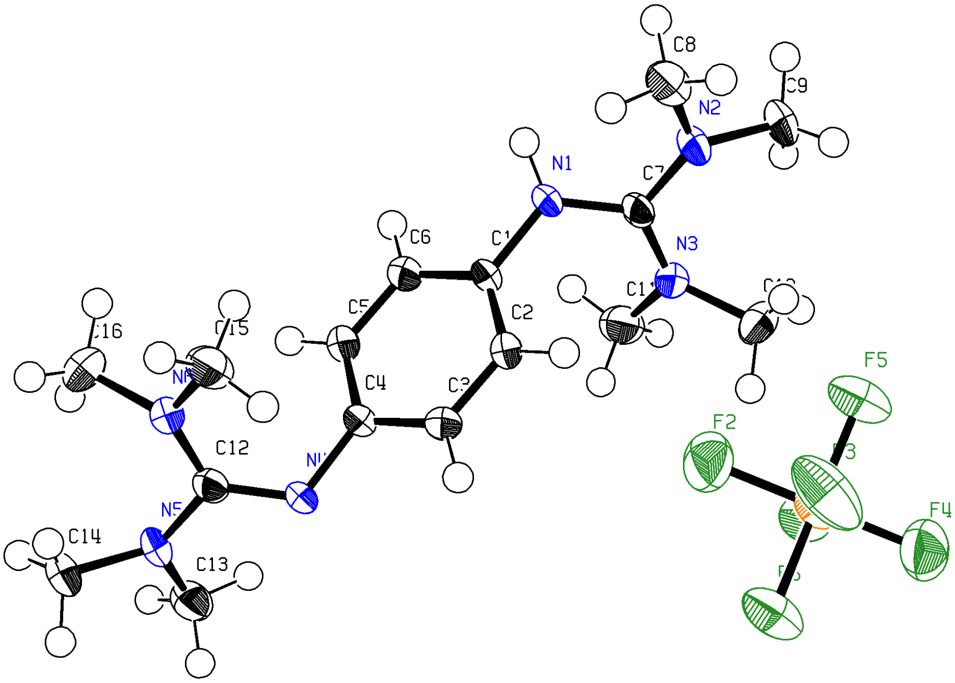

Figure S5. ORTEP of monoprotonated **1,4-btmgbH[PF$_6$]**. Thermal ellipsoids are shown at 80% probability. See refinement data tables **S14-19** starting on **S34**.

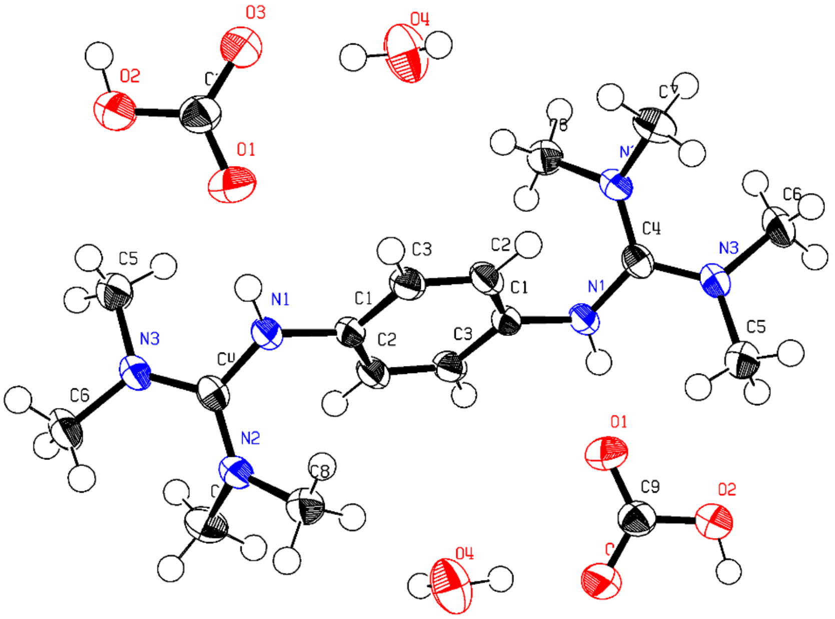

Figure S6. ORTEP of **1,4-btmgbH$_2$(HCO$_3^-$)$_2$•(H$_2$O)$_2$**, ellipsoids are shown at 80% probability. See refinement data tables **S20-26** starting on **S43**.

All of the CVs were carried out at room temperature. The scan starting points and direction are indicated by the arrows.

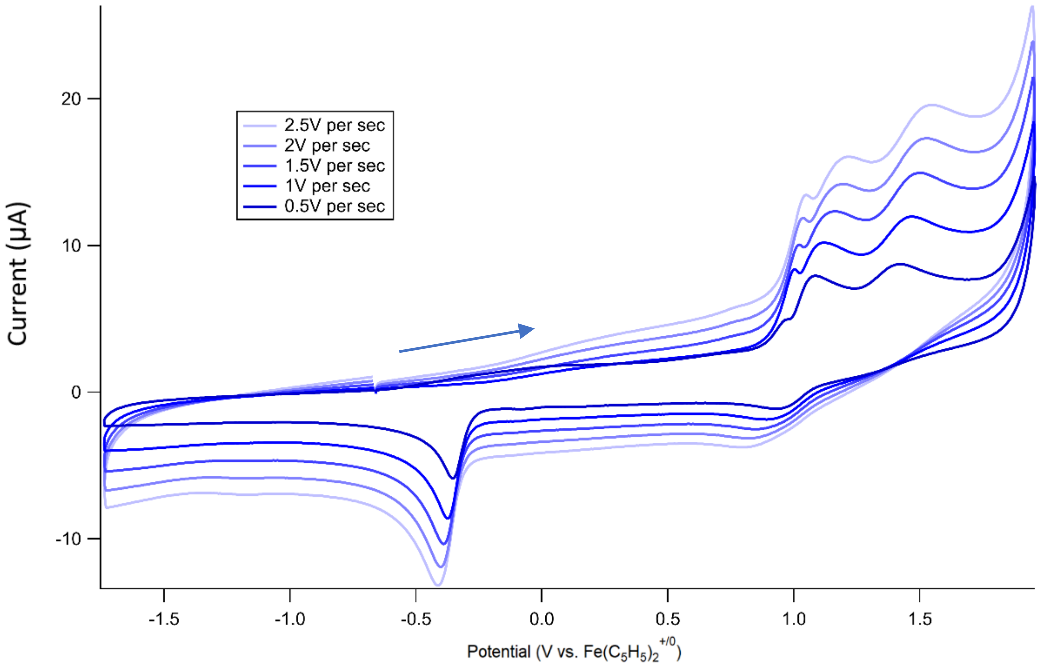

Figure S7. 1 mM 1,4-btmgbH$_2$[PF$_6$]$_2$ in MeCN with 100 mM TBAPF$_6$, at varied scan rates.

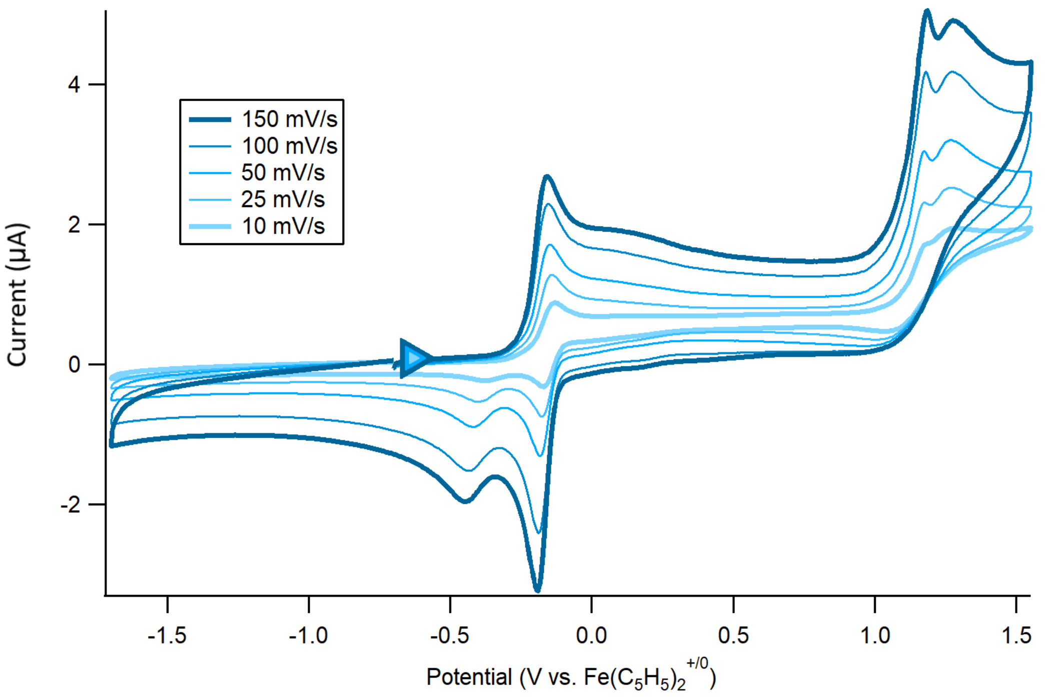

Figure S8. 1 mM **1,4-btmgbH[PF$_6$]** in MeCN with 100 mM TMAPF$_6$, varied scan rate.

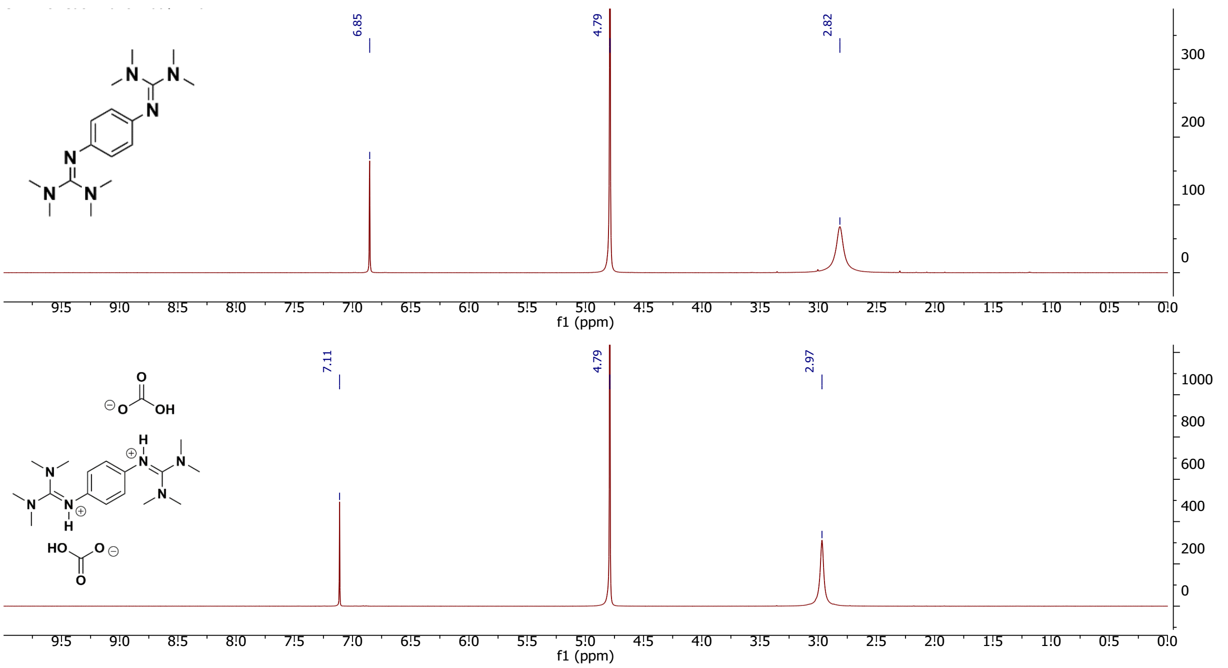

Figure S9. ^{1}H NMR of **1,4-btmgb** in D_2O, 600 MHz, 298 K, under 1 atm N_2 (top) and under 1 atm of CO_2 (bottom).

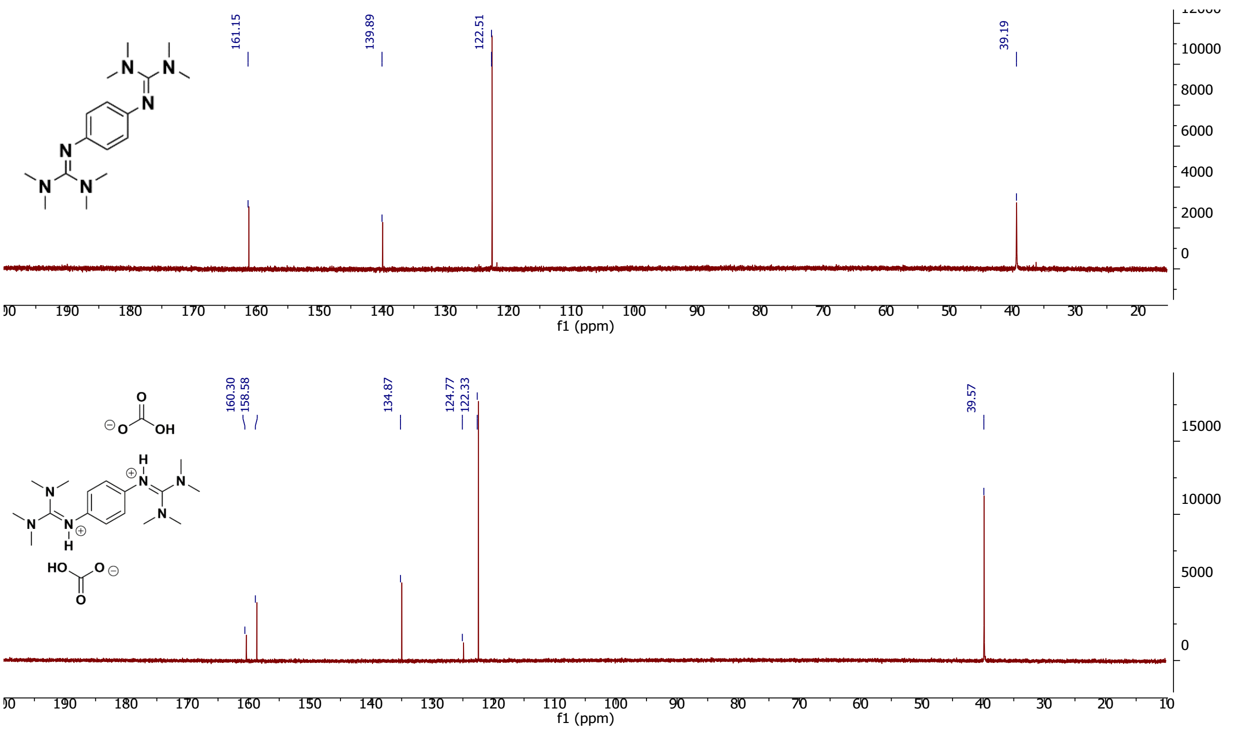

Figure S10. $^{13}C\{^{1}H\}$ NMR of **1,4-btmgb** in D_2O, 151 MHz, 298 K, under 1 atm N_2 (top) and under 1 atm CO_2 (bottom).

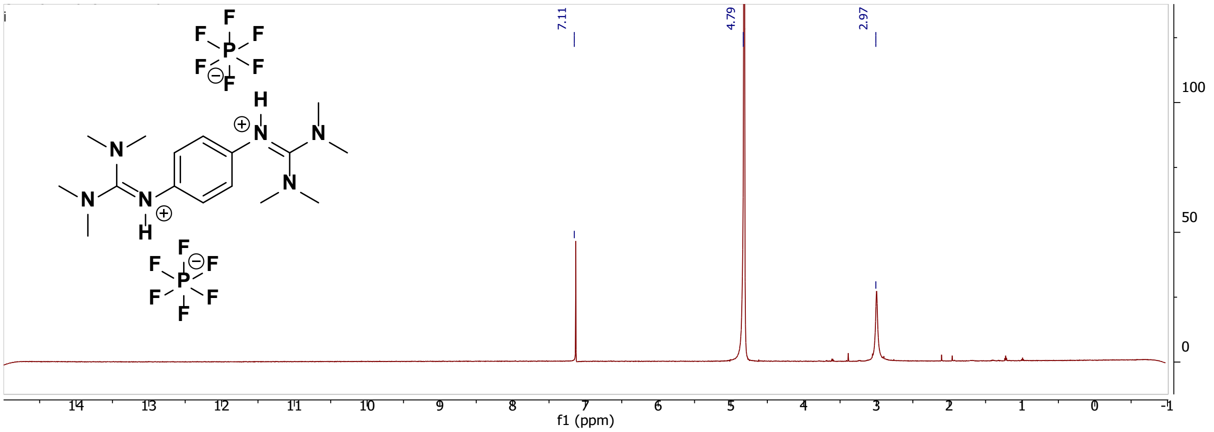

Figure S11. ^{1}H NMR of **1,4-btmgbH$_2$[PF$_6$]$_2$** in D_2O, 600 MHz, 298 K.

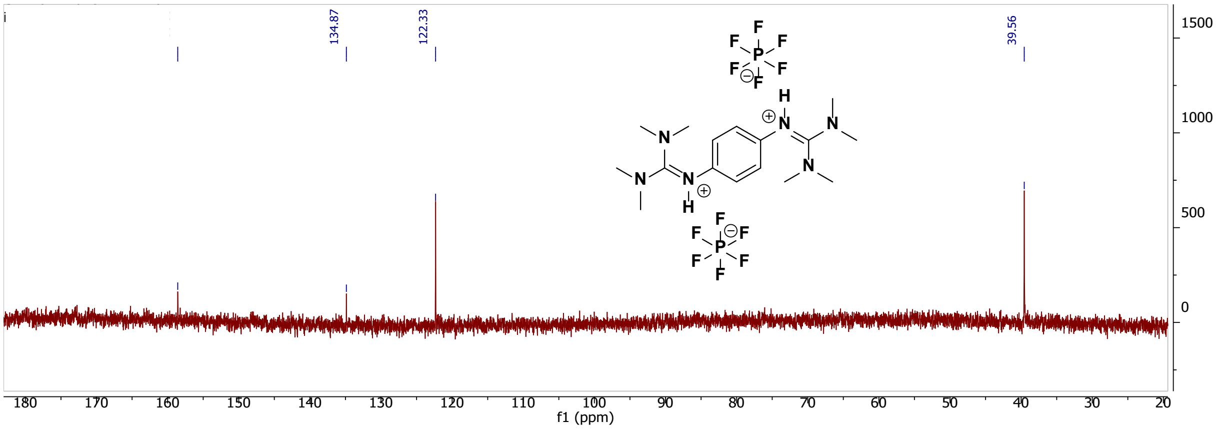

Figure S12. $^{13}C\{^{1}H\}$ of **1,4-btmgbH$_2$[PF$_6$]$_2$** in D_2O, 151 MHz, 298 K.

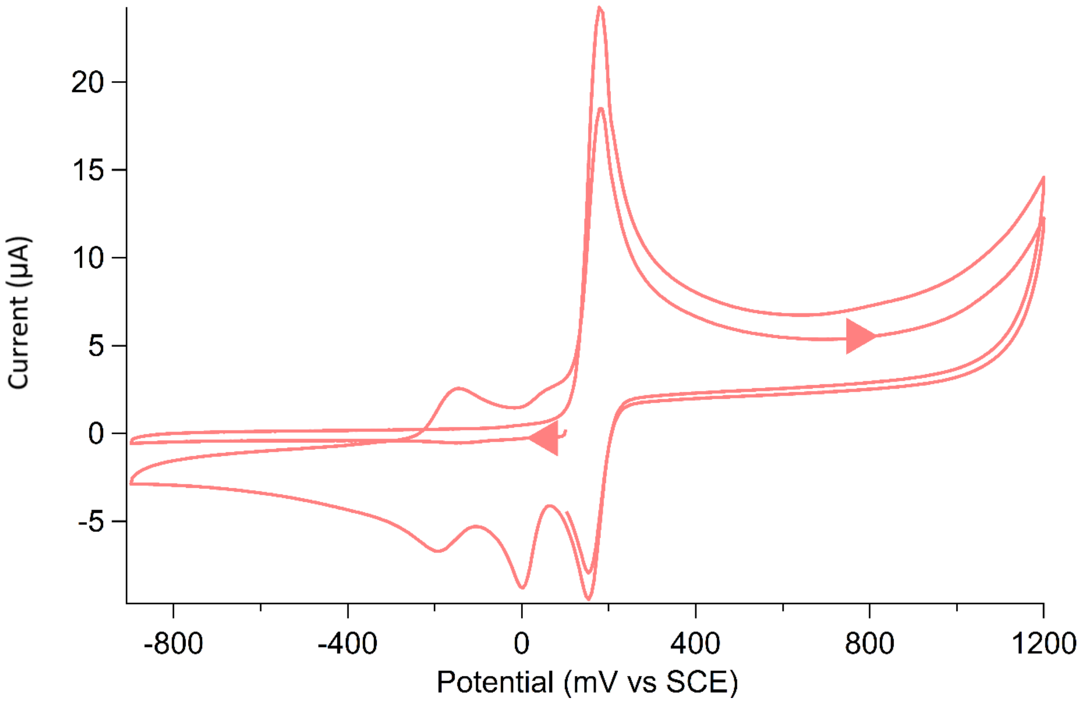

Figure S13. Cyclic voltammetry investigating the reduction events at 0 and -0.18 V vs. SCE of **1,4-btmgbH^{+}** in water by scanning reductively first before scanning again after the main redox peak at 0.2 V vs SCE (**1,4-btmgb/btmgb^{2+}**). 1 mM **1,4-btmgb** in water with 100 mM KCl at 250 mV/s, at pH 11.72.

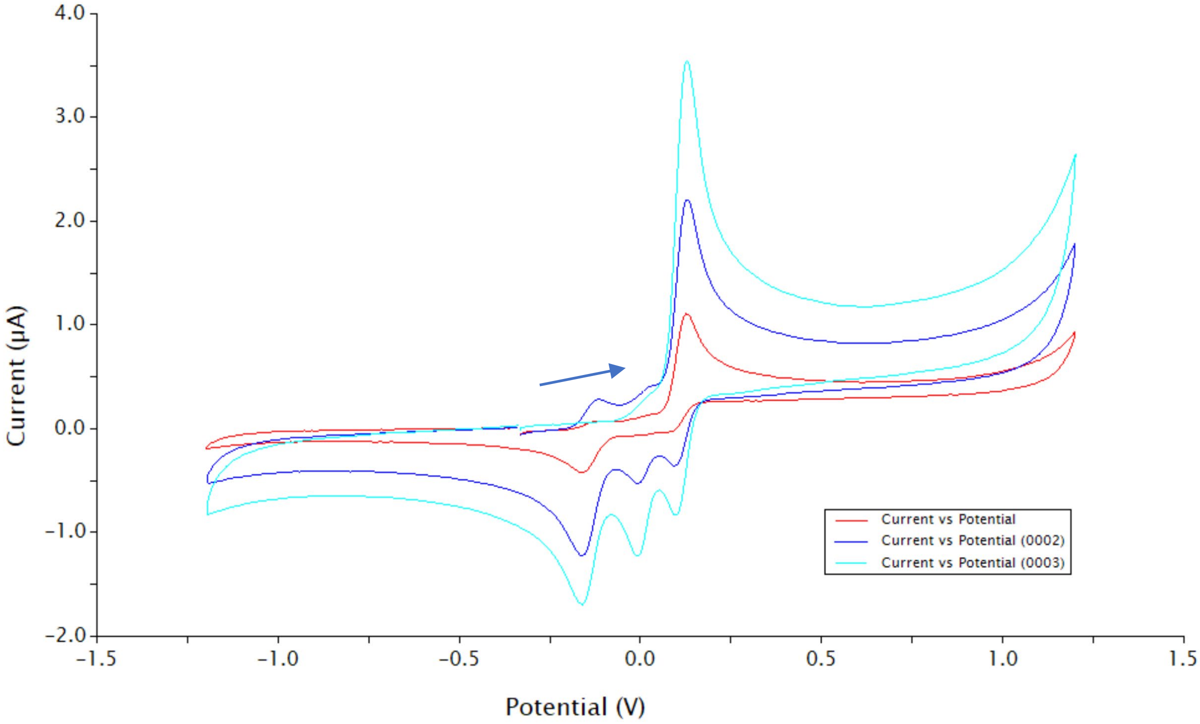

Figure S14. Cyclic voltammetry at varied scan rates of 1mM **1,4-btmgb** vs. SCE, dissolved in water with 100 mM KCl at a pH of 11.72 (no additional acid or base). Scan rates 10 (red), 50 (blue), and 100 mV/s (cyan)

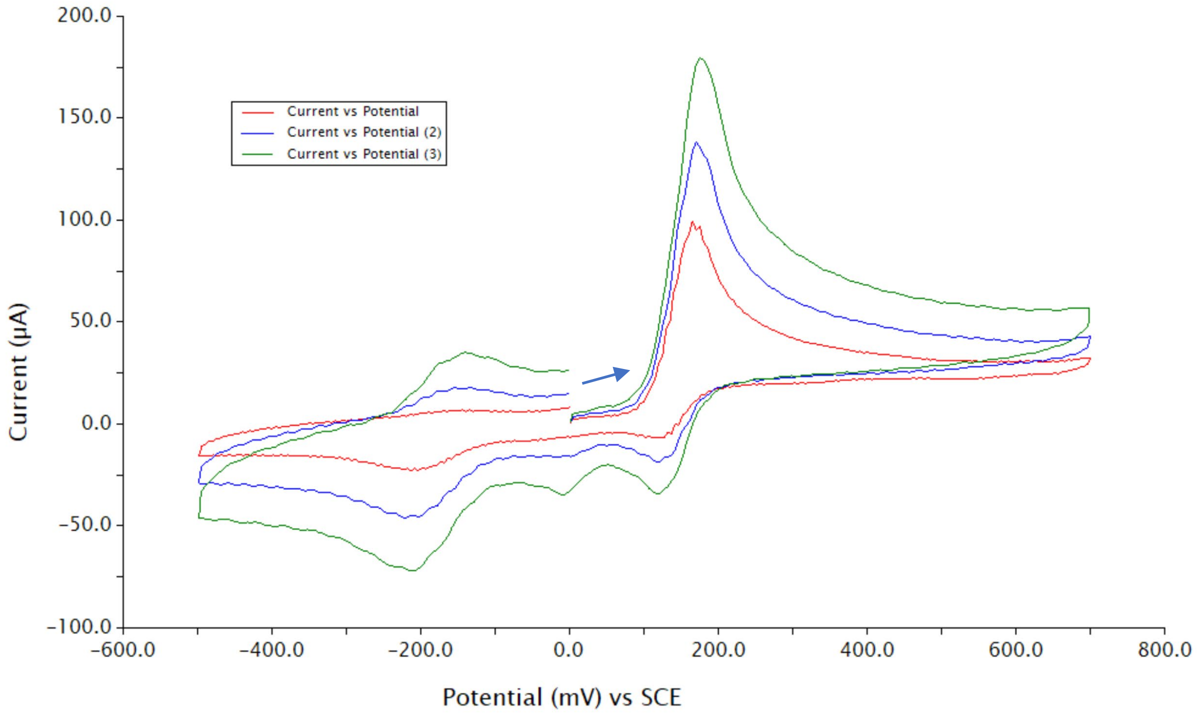

Figure S15. Cyclic voltammetry of **1,4-btmgb** in water with 100 mM KCl and NaOH to adjust to a pH of 12.84. Scan rates 200 (red), 400 (blue), and 700 mV/s (green).

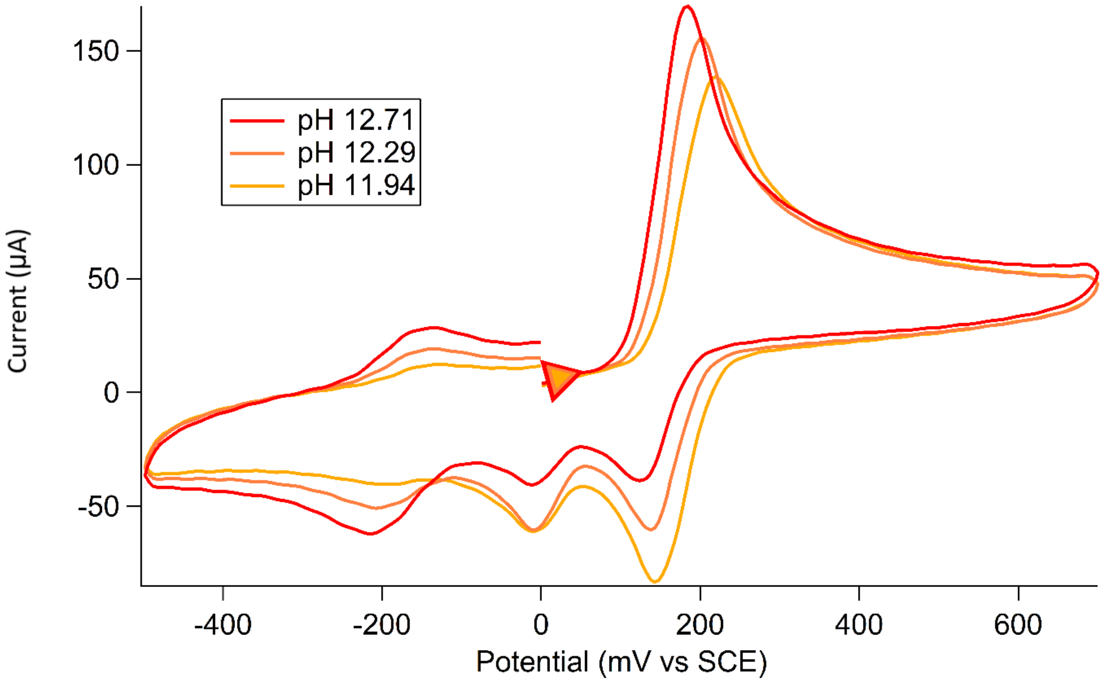

Figure S16. Cyclic voltammetry of 1 mM **1,4-btmgb** vs SCE in water with 100 mM KCl at varied alkaline conditions by the additions of concentrated NaOH, scan rate = 400 mV/s.

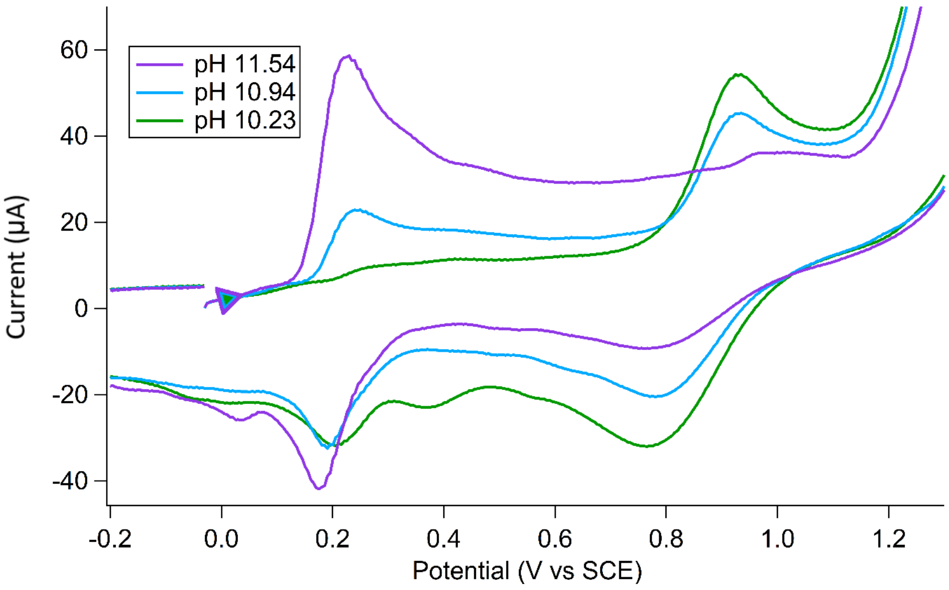

Figure S17. Cyclic voltammetry of 1 mM **1,4-btmgb** in water with 100 mM KCl, scan rate of 100 mV/s. Varied solution pH with additions of 1 M HCl.

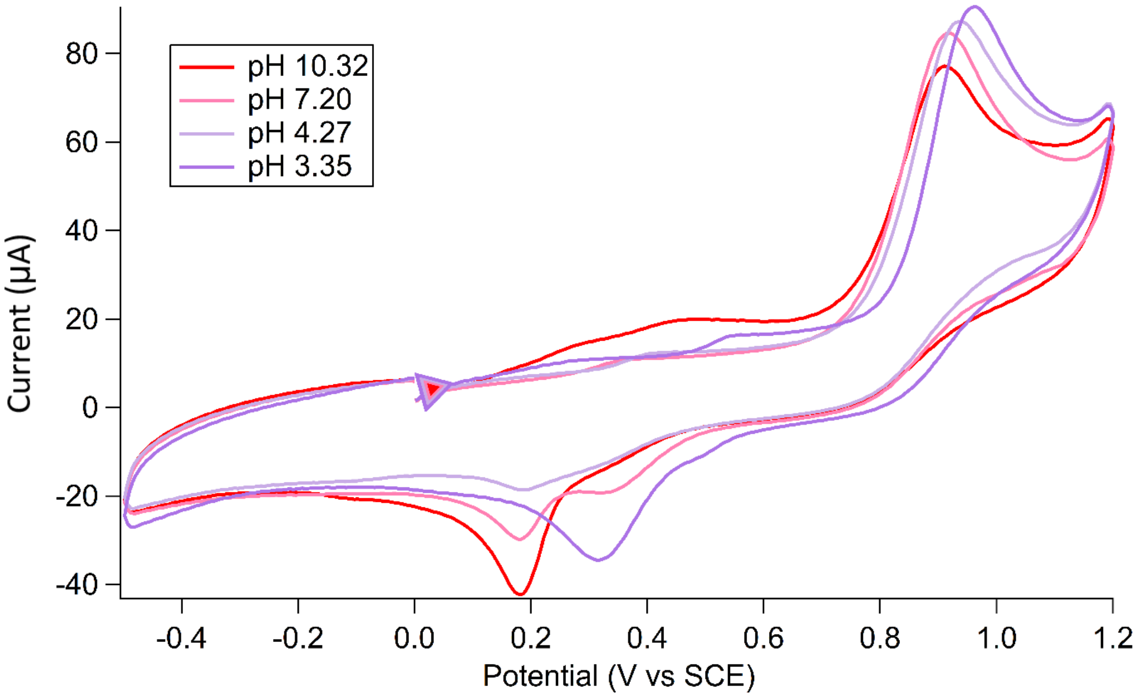

Figure S18. Cyclic voltammetry of 1 mM **1,4-btmgb** (protonated to **1,4-btmgbH$_2$$^{2+}$** by solution pH) in water with 100 mM KCl, scan rate 400 mV/s. Varied solution pH with additions of 1 M HCl.

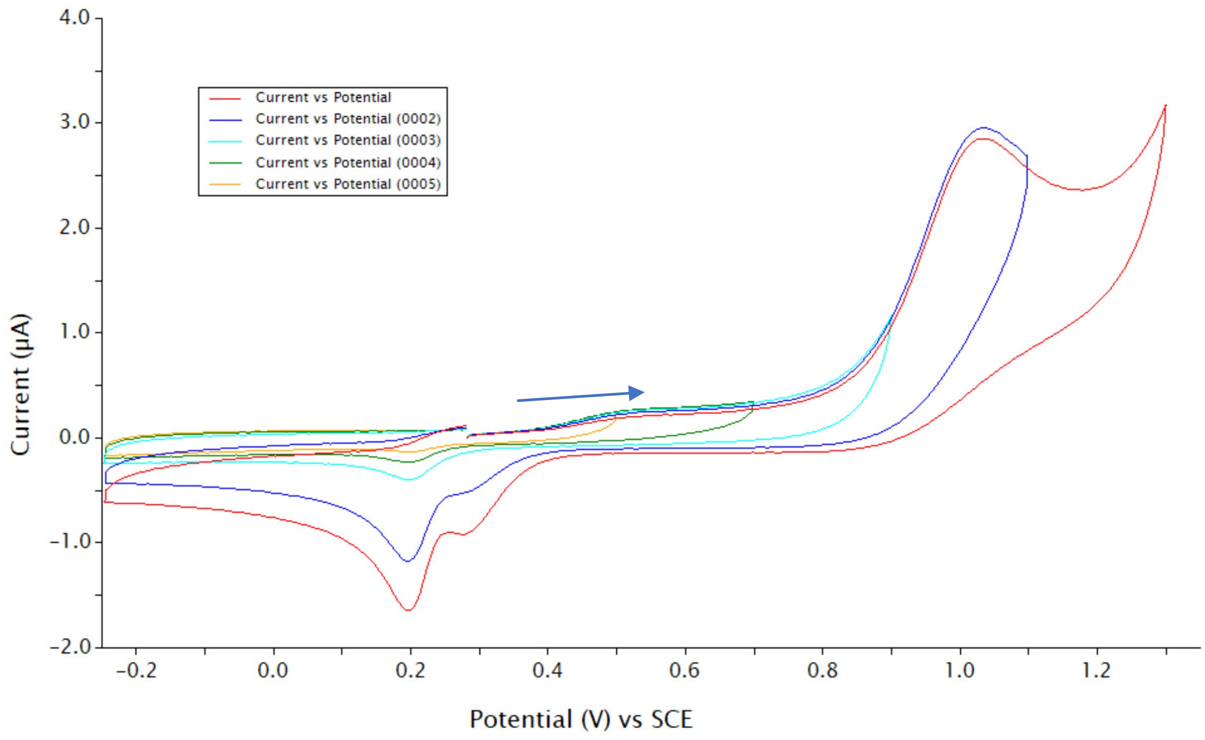

Figure S19. Cyclic voltammetry of 1 mM **[1,4-btmgbH$_2$]Cl$_2$** for comparative reference of protonated species. In water with 100 mM KCl – pH 9.88, scan rate 250 mV/s. Varied scan window.

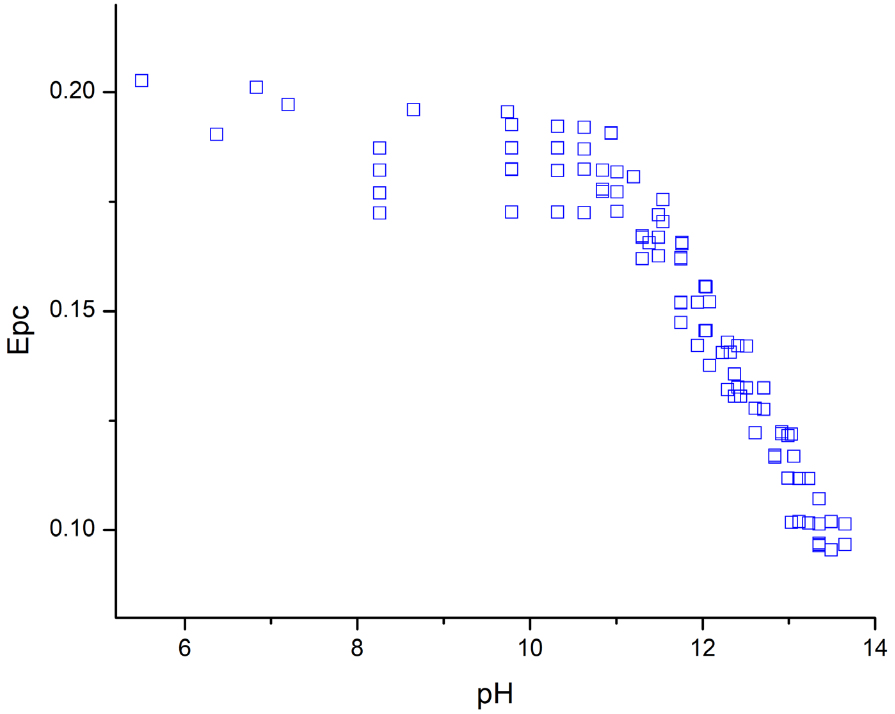

Figure S20. pH measurements *vs.* E_{pc} values from pH 13.79 to 2.52, taken from an initial solution of 1 mM btmgb dissolved in water with 100 mM KCl and titrated with 1 M NaOH and 1 M HCl or 1 M acetic acid. Collective data from varied scan rates of 200 mV, 400 mV, and 700 mV.

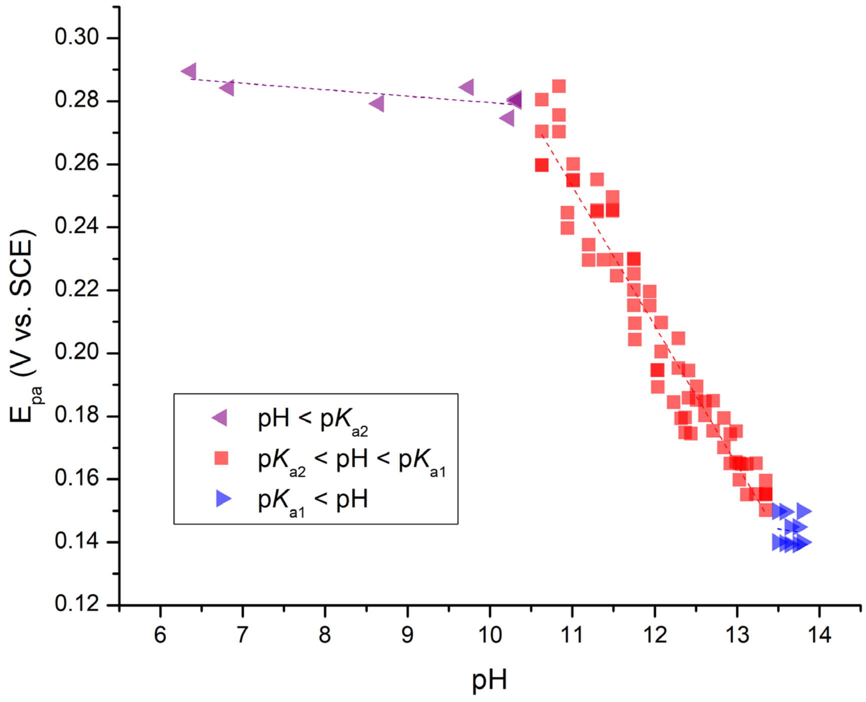

Figure S21. pH measurements *vs.* E_{pa} values from pH 13.79 to 2.52, taken from an initial solution of 1 mM btmgb dissolved in water with 100 mM KCl and titrated with 1 M NaOH and 1 M HCl or 1 M acetic acid. Collective data from varied scan rates of 200 mV, 400 mV, and 700 mV.

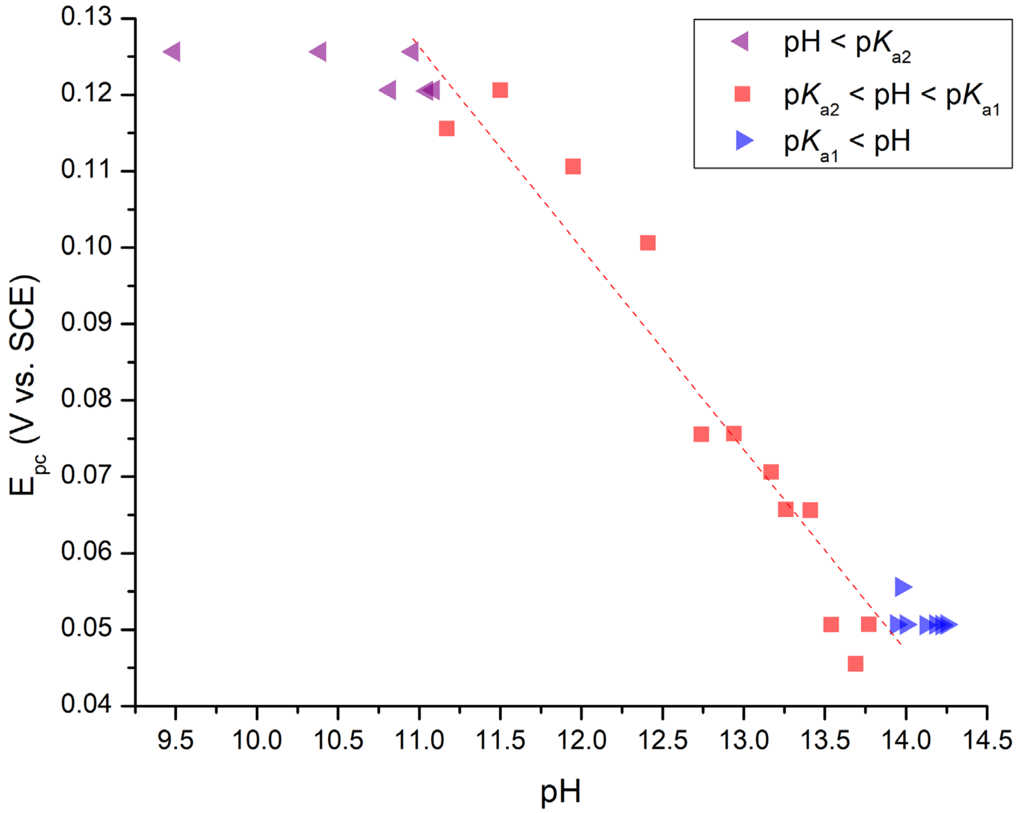

Figure S22. pH measurements *vs.* E_{pc} (from a pairing CV with DPV experiments) from pH 14.12 to 9.49, taken from an initial solution of 2 mM btmgb dissolved in water with 200 mM KCl and titrated with 0.1 M, 1 M, or 50 wt% NaOH and 0.1 M or 1 M HCl. CV's were taken with a scan rate of 500 mV/s.

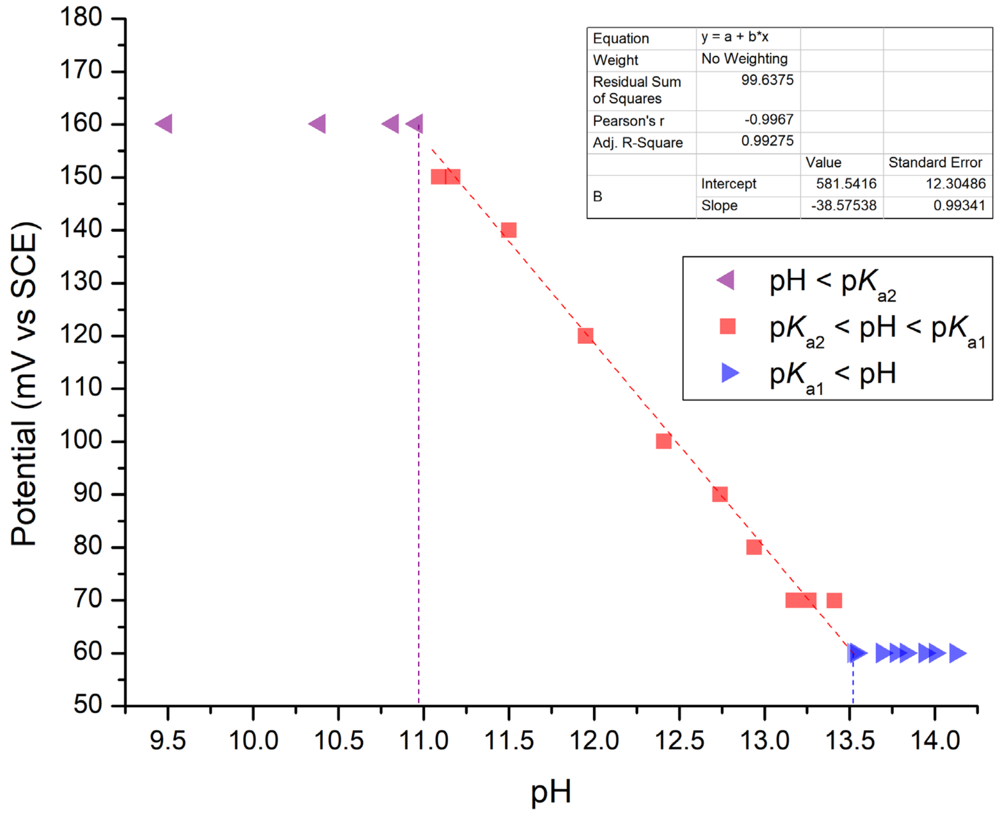

Figure S23. pH measurements *vs.* E_p, differential-pulse voltammetry (DPV) data from pH 14.12 to 9.49, taken from an initial solution of 2 mM btmgb dissolved in water with 200 mM KCl and titrated with 0.1 M, 1 M, or conc. NaOH and 0.1 M or 1 M HCl.

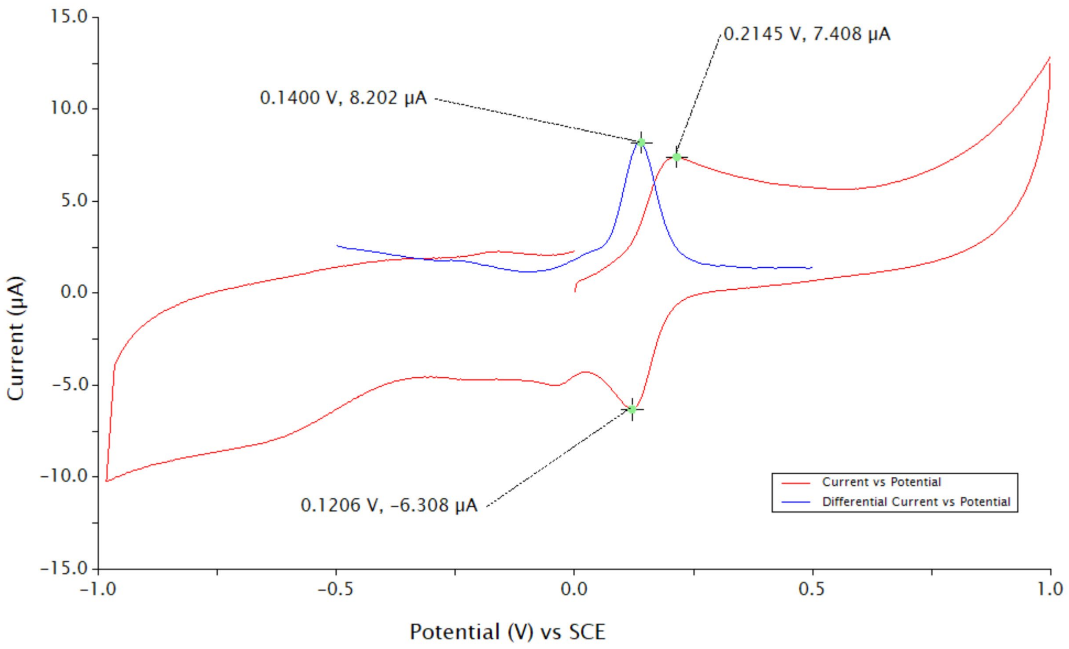

Figure S24. Example work up of CV and DPV data, collecting E_{pc}, E_{pa}, and E_p. CV and DPV of 1,4 btmgb, initial concentration 2 mM in a 200 mM KCl solution, pH adjusted to 11.5 pH with a mix of 0.1 M NaOH and 0.1 M HCl. CV scan rate of 500 mV/s.

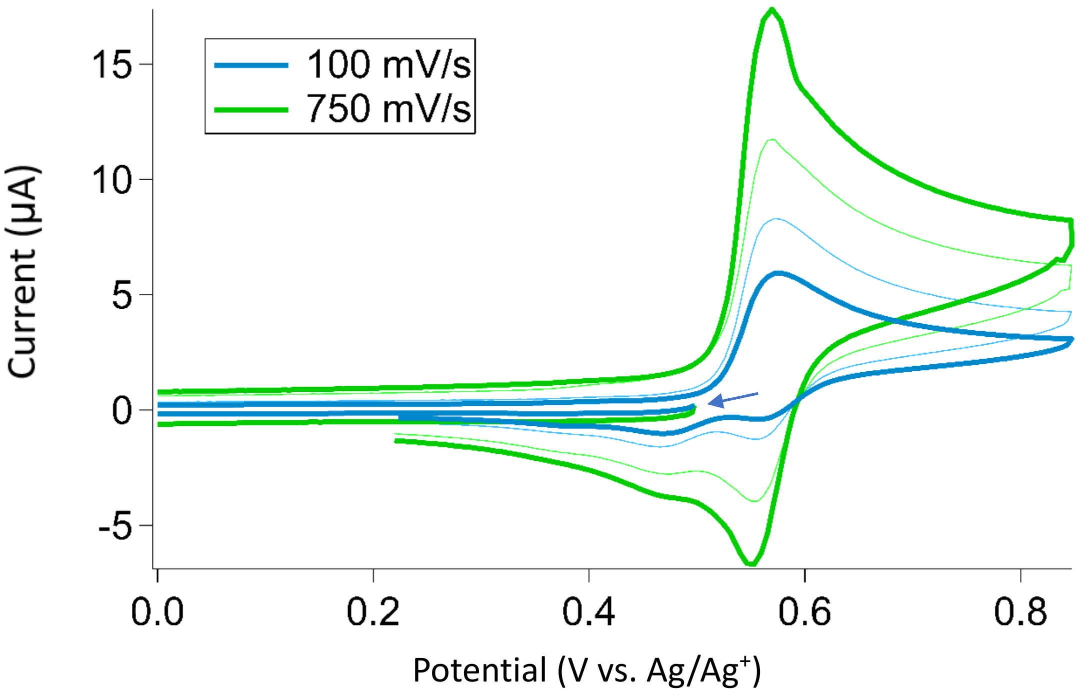

Figure S25. Cyclic voltammetry of 1 mM **1,4-btmgb** in MeOH with 100 mM NaBr referenced *vs* Ag/Ag$^+$.

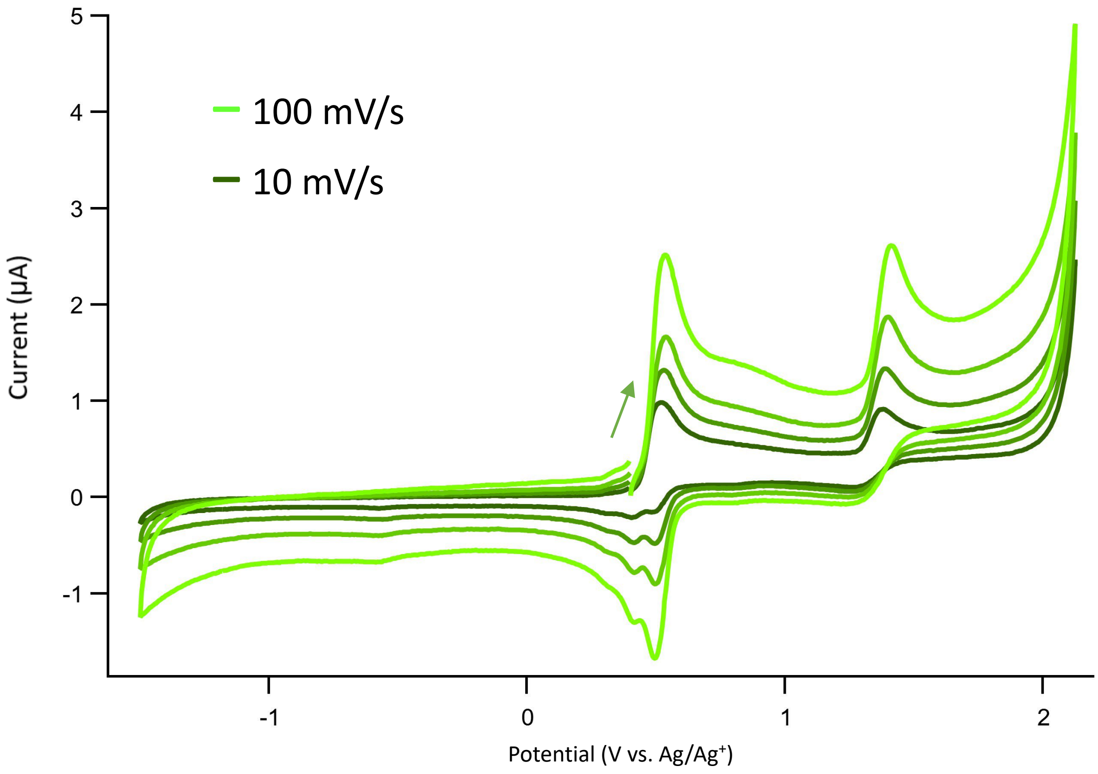

Figure S26. Cyclic voltammetry of 1mM **1,4-btmgb** in EtOH with 100 mM $NaClO_4$ referenced *vs* Ag/Ag$^+$.

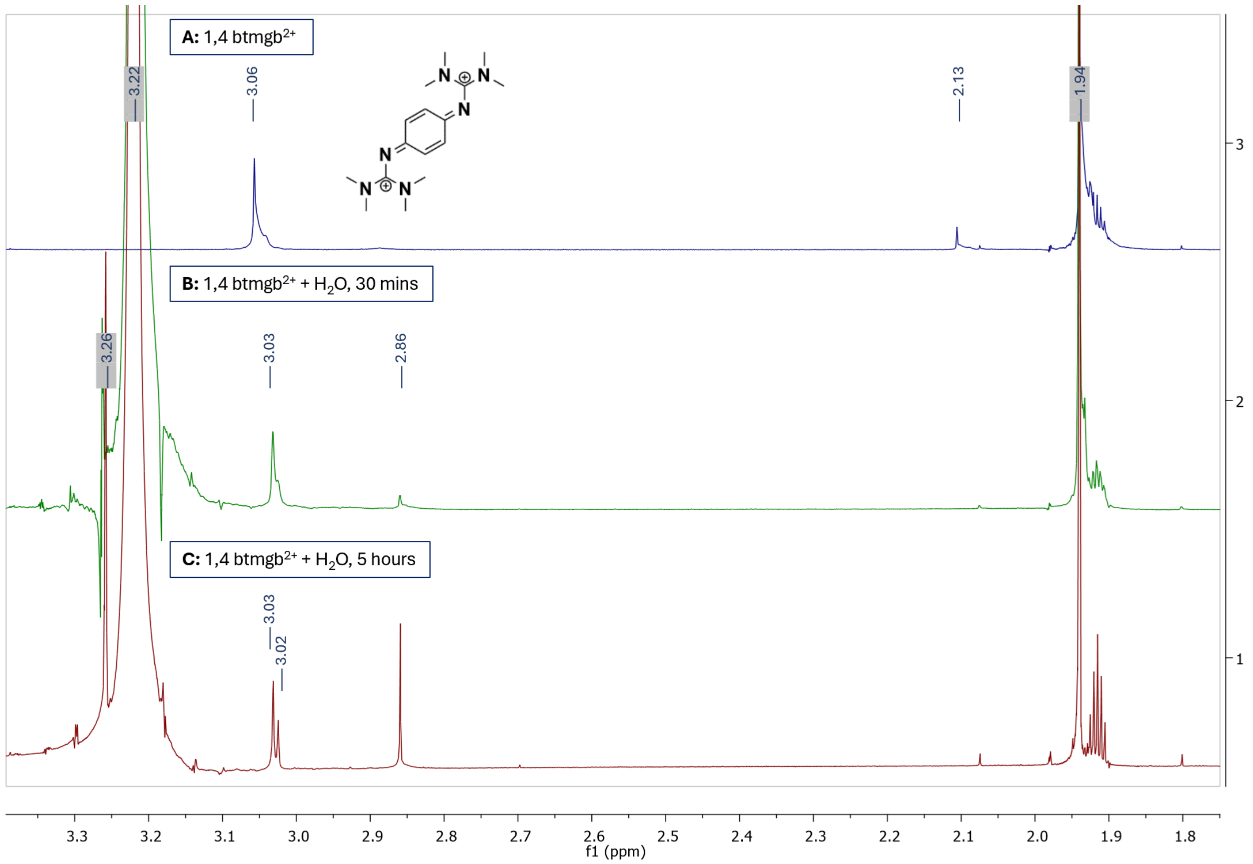

Figure S27. ^{1}H NMR (500 Hz, 293 K) spectra of the oxidized species **1,4-btmgb[BF$_4$]$_2$** in CD_3CN before (**A**) and after (**B** & **C**) water addition, methyl region. The high amounts of water added to this solution result in the residual water peak that is typically at 2.13 ppm (in CD_3CN) with respect to the solvent residual peak to shift downfield to 3.22 ppm due to H-bonding.

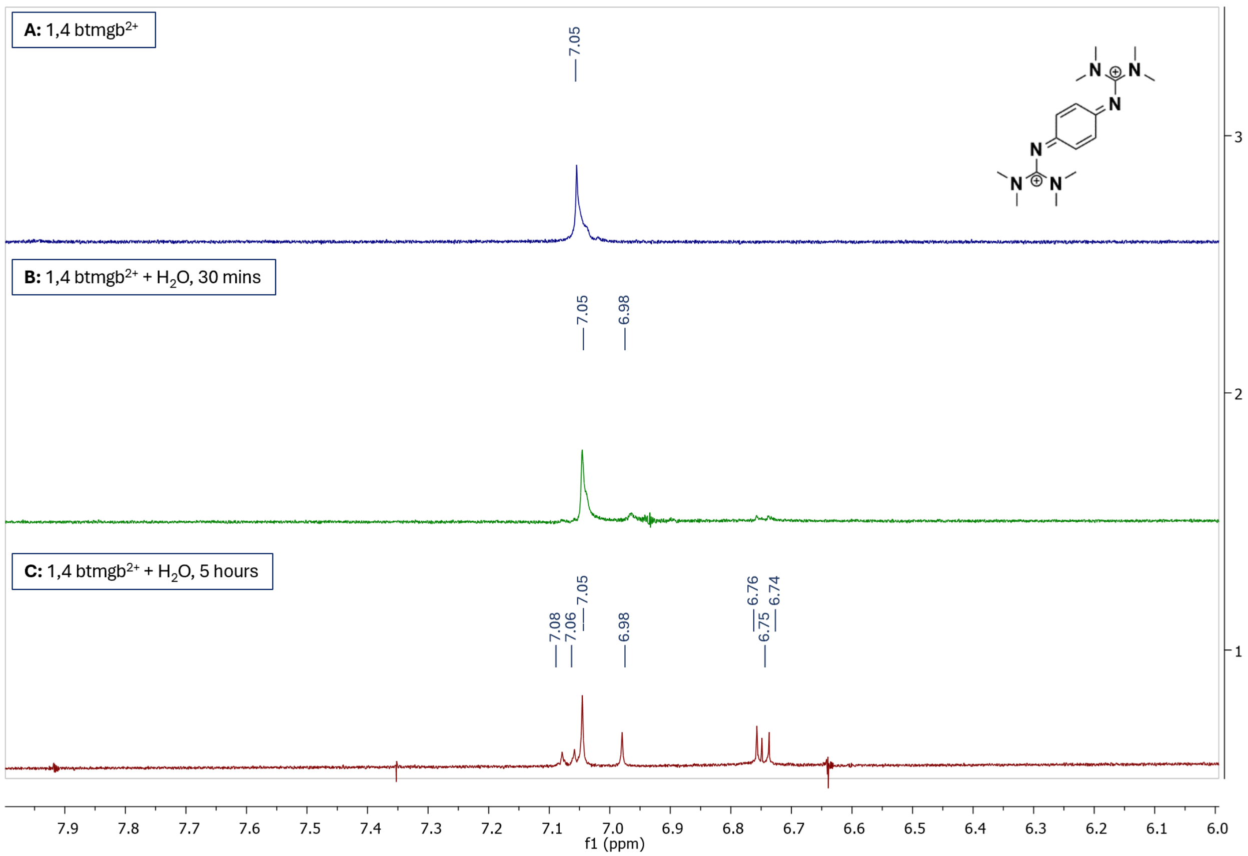

Figure S28. ^{1}H NMR (500 Hz, 293 K) spectra of **1,4-btmgb[BF$_4$]$_2$** in CD_3CN before (**A**) and after (**B** & **C**) water addition, aromatic region

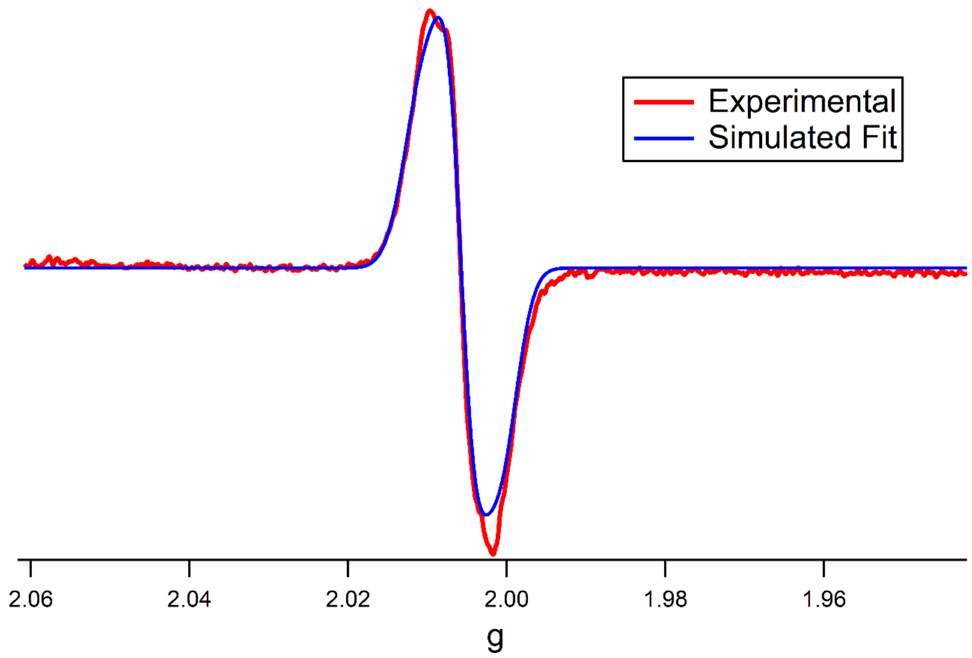

Figure S29. EPR spectrum of 2 mM 1,4 btmgb oxidized for 10 mins, measured at 77K, microwave frequency = 9.43 GHz.

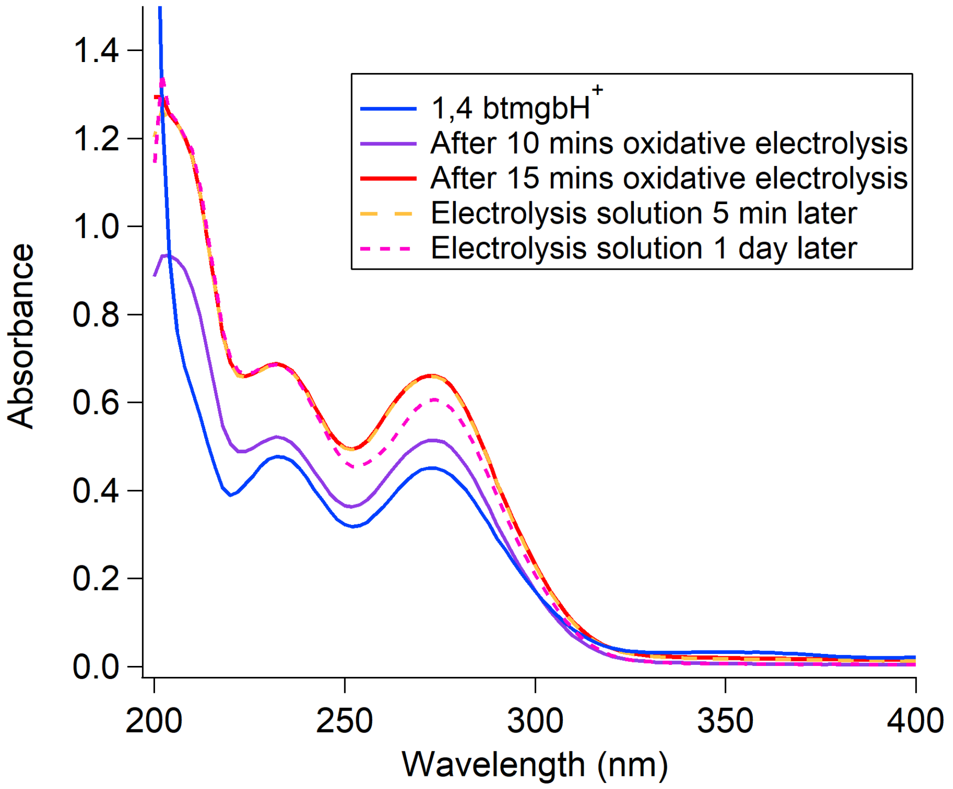

Figure S30. UV-Vis spectra before and after controlled oxidative electrolysis of 1 mM 1,4 btmgbH$^+$ in water with 100 mM KCl. Oxidation held at 0.5 V vs. SCE. Sample diluted for UV-Vis measurement.

Solubility of 1,4-btmgb was found using a shake flask method where a saturated solution of 1,4-btmgb in water was shaken in a sonicator for several minutes and allowed to stir overnight for maximum solubility. A 2mM solution of 1,4-btmgb in water was also prepared and diluted into various concentrations to create a calibration curve (where a ∈ = 24.77). The following day, UV-Vis spectra were taken of a filtered and then diluted sample from the saturated 1,4-btmgb solution under the exposure or absence of CO_2. It should be noted that discrepancies in the absorbances to solution concentrations observed between other experiments and this solubility experiment are due to the time needed for 1,4-btmgb to fully dissolve into solution.

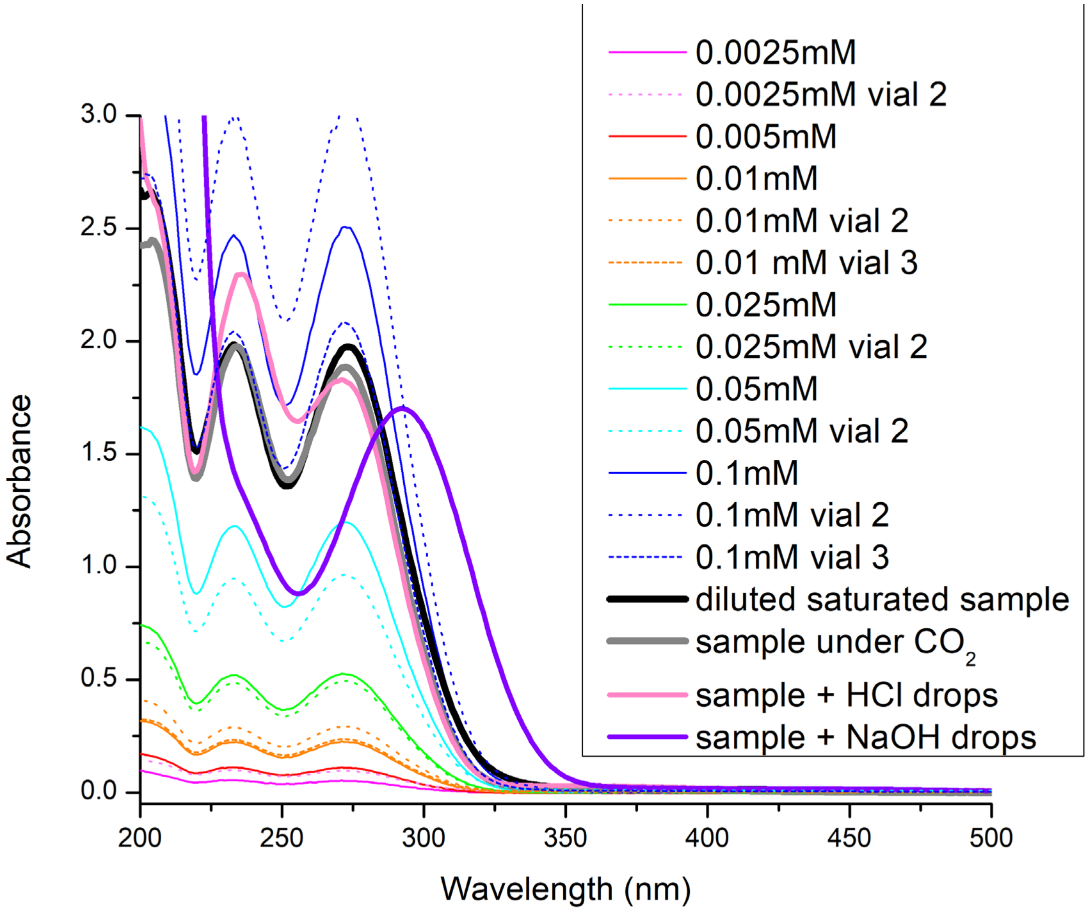

Figure S31. UV-Vis spectra of 1,4-btmgb at different concentrations in water.

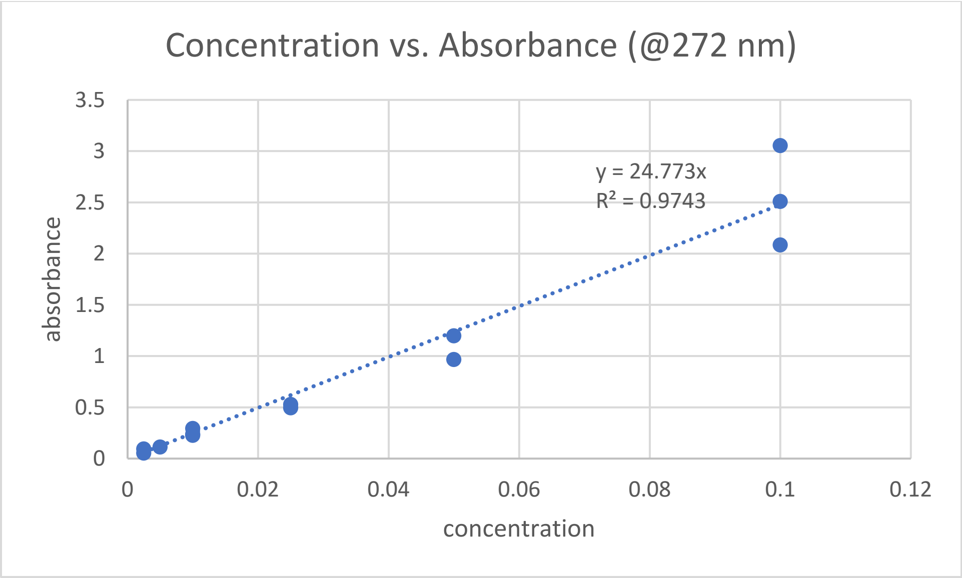

Figure S32. Concentration vs. Absorbance of 1,4-btmgb in water.

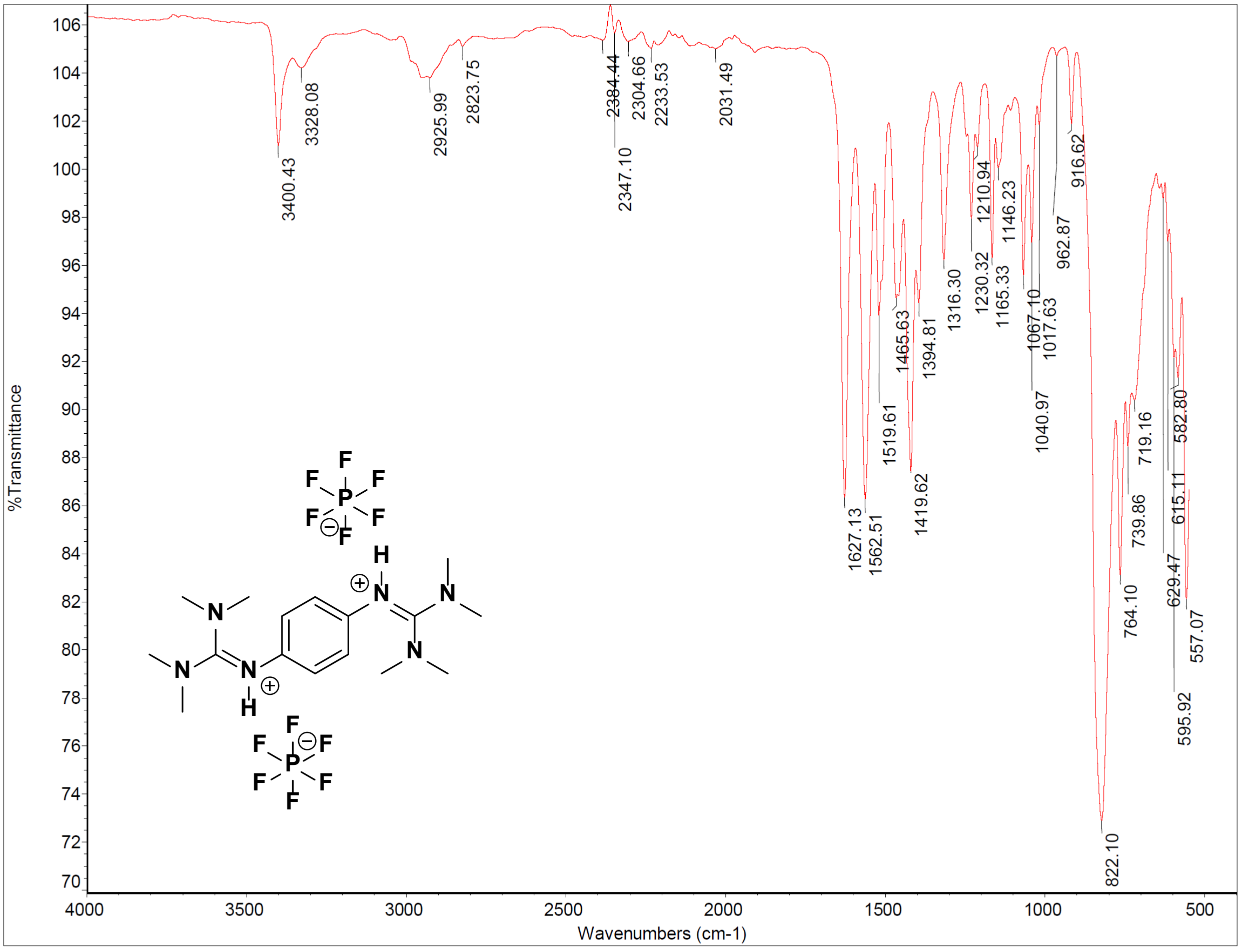

Figure S33. FT-IR spectrum of 1,4-btmgbH_2[PF_6]$_2$.

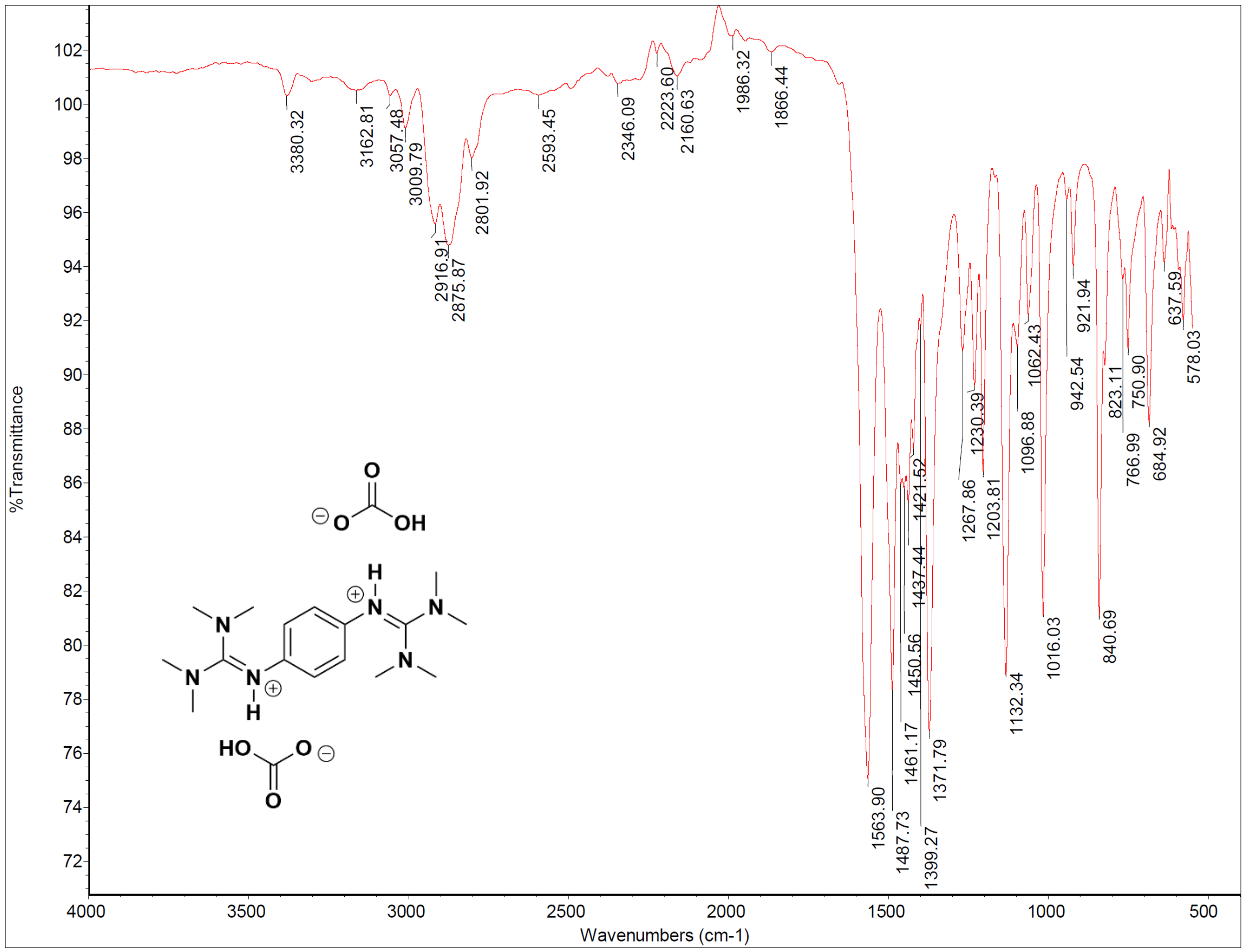

Figure S34. FT-IR spectrum of 1,4-btmgb$H_2[CO_3H]_2$.

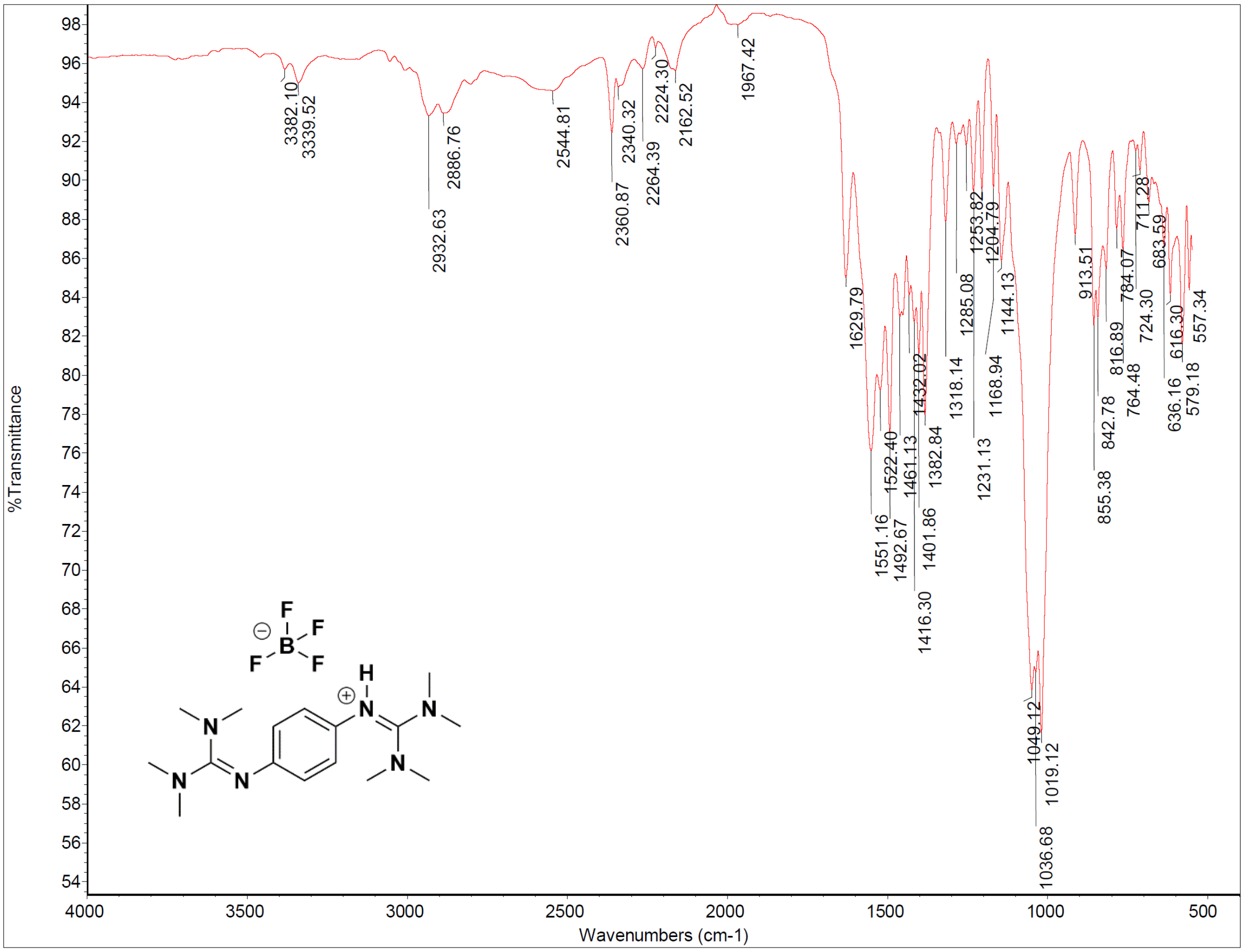

Figure S35. FT-IR spectrum of 1,4-btmgbH[BF_4].

Table S1. Crystal data and structure refinement for **1,4-btmgbH$_2$(HCO$_3^-$)$_2$.**
(Structure in main text Figure 2)

Empirical formula	[C_{16} H_{30} N_6] [C H O_3]$_2$	
Formula weight	428.49	
Temperature	93(2) K	
Wavelength	0.71073 Å	
Crystal system	Monoclinic	
Space group	$P2_1/n$	
Unit cell dimensions	a = 9.7836(14) Å	α = 90°.
	b = 8.1140(12) Å	β = 96.738(2)°.
	c = 13.4892(19) Å	γ = 90°.
Volume	1063.4(3) Å^3	
Z	2	
Density (calculated)	1.338 Mg/m^3	
Absorption coefficient	0.101 mm^{-1}	
F(000)	460	
Crystal color	colorless	
Crystal size	0.228 x 0.201 x 0.082 mm^3	
Theta range for data collection	2.441 to 30.544°	
Index ranges	$-13 \le h \le 13$, $-11 \le k \le 11$, $-19 \le l \le 19$	
Reflections collected	25041	
Independent reflections	3242 [R(int) = 0.0527]	
Completeness to theta = 25.242°	100.0 %	
Absorption correction	Semi-empirical from equivalents	
Max. and min. transmission	0.7461 and 0.6978	
Refinement method	Full-matrix least-squares on F^2	
Data / restraints / parameters	3242 / 0 / 200	
Goodness-of-fit on F^2	1.046	
Final R indices [I>2sigma(I) = 2503 data]	R1 = 0.0402, wR2 = 0.0916	
R indices (all data, ? Å)	R1 = 0.0614, wR2 = 0.1008	
Largest diff. peak and hole	0.353 and -0.213 e.Å^{-3}	

Table S2. Atomic coordinates (x 10^4) and equivalent isotropic displacement parameters (Å^2 x 10^3) for **1,4-btmgbH$_2$(HCO$_3^-$)$_2$**. U(eq) is defined as one third of the trace of the orthogonalized U^{ij} tensor.

	x	y	z	U(eq)
O(1)	5889(1)	7546(1)	3310(1)	20(1)
O(2)	4349(1)	9290(1)	3767(1)	22(1)
O(3)	6207(1)	8657(1)	4841(1)	20(1)
C(9)	5543(1)	8454(1)	3987(1)	15(1)
N(1)	8639(1)	6975(1)	3474(1)	13(1)
N(2)	10480(1)	8276(1)	2841(1)	15(1)
N(3)	8514(1)	7504(1)	1784(1)	14(1)
C(1)	9362(1)	6006(1)	4246(1)	12(1)

C(2)	9007(1)	6143(1)	5216(1)	13(1)
C(3)	10352(1)	4863(1)	4029(1)	13(1)
C(4)	9229(1)	7572(1)	2690(1)	12(1)
C(5)	10982(1)	9028(2)	3798(1)	20(1)
C(6)	11480(1)	8244(2)	2112(1)	21(1)
C(7)	7375(1)	6333(2)	1559(1)	19(1)
C(8)	8727(1)	8653(2)	978(1)	20(1)

Table S3. Bond lengths [Å] and angles [°] for **1,4-btmgbH$_2$(HCO$_3^-$)$_2$**.

O(1)-C(9)	1.2505(14)
O(2)-C(9)	1.3542(13)
O(2)-H(2)	0.950(19)
O(3)-C(9)	1.2649(14)
N(1)-C(4)	1.3529(14)
N(1)-C(1)	1.4241(13)
N(1)-H(1)	0.912(18)
N(2)-C(4)	1.3449(14)
N(2)-C(5)	1.4601(14)
N(2)-C(6)	1.4658(15)
N(3)-C(4)	1.3358(13)
N(3)-C(8)	1.4651(15)
N(3)-C(7)	1.4689(15)
C(1)-C(2)	1.3969(15)
C(1)-C(3)	1.3970(15)
C(2)-C(3)#1	1.3946(15)
C(2)-H(2A)	0.958(14)
C(3)-C(2)#1	1.3946(15)
C(3)-H(3)	0.950(13)
C(5)-H(5A)	0.959(15)
C(5)-H(5B)	0.990(17)
C(5)-H(5C)	0.992(16)
C(6)-H(6A)	0.985(16)
C(6)-H(6B)	0.989(15)

C(6)-H(6C)	0.984(17)
C(7)-H(7A)	0.994(15)
C(7)-H(7B)	0.981(16)
C(7)-H(7C)	0.982(15)
C(8)-H(8A)	0.998(15)
C(8)-H(8B)	0.977(16)
C(8)-H(8C)	0.967(15)
C(9)-O(2)-H(2)	109.1(11)
O(1)-C(9)-O(3)	126.04(10)
O(1)-C(9)-O(2)	115.78(10)
O(3)-C(9)-O(2)	118.18(10)
C(4)-N(1)-C(1)	123.25(9)
C(4)-N(1)-H(1)	117.1(11)
C(1)-N(1)-H(1)	119.2(11)
C(4)-N(2)-C(5)	121.10(9)
C(4)-N(2)-C(6)	123.92(9)
C(5)-N(2)-C(6)	114.81(9)
C(4)-N(3)-C(8)	123.20(10)
C(4)-N(3)-C(7)	121.26(9)
C(8)-N(3)-C(7)	115.37(9)
C(2)-C(1)-C(3)	120.19(9)
C(2)-C(1)-N(1)	118.97(9)
C(3)-C(1)-N(1)	120.74(9)
C(3)#1-C(2)-C(1)	119.86(10)
C(3)#1-C(2)-H(2A)	120.7(8)
C(1)-C(2)-H(2A)	119.5(8)
C(2)#1-C(3)-C(1)	119.96(9)
C(2)#1-C(3)-H(3)	119.0(8)
C(1)-C(3)-H(3)	121.0(8)
N(3)-C(4)-N(2)	121.70(10)
N(3)-C(4)-N(1)	118.36(9)
N(2)-C(4)-N(1)	119.89(9)
N(2)-C(5)-H(5A)	110.1(9)
N(2)-C(5)-H(5B)	108.7(9)
H(5A)-C(5)-H(5B)	109.9(13)
N(2)-C(5)-H(5C)	111.0(9)

H(5A)-C(5)-H(5C)	108.9(13)
H(5B)-C(5)-H(5C)	108.2(13)
N(2)-C(6)-H(6A)	108.4(9)
N(2)-C(6)-H(6B)	110.9(8)
H(6A)-C(6)-H(6B)	110.0(12)
N(2)-C(6)-H(6C)	110.8(9)
H(6A)-C(6)-H(6C)	107.7(13)
H(6B)-C(6)-H(6C)	108.9(13)
N(3)-C(7)-H(7A)	110.1(9)
N(3)-C(7)-H(7B)	107.2(9)
H(7A)-C(7)-H(7B)	110.3(12)
N(3)-C(7)-H(7C)	109.8(8)
H(7A)-C(7)-H(7C)	109.5(12)
H(7B)-C(7)-H(7C)	110.0(12)
N(3)-C(8)-H(8A)	107.1(9)
N(3)-C(8)-H(8B)	111.1(9)
H(8A)-C(8)-H(8B)	108.9(13)
N(3)-C(8)-H(8C)	110.4(9)
H(8A)-C(8)-H(8C)	108.7(12)
H(8B)-C(8)-H(8C)	110.5(12)

Symmetry transformations used to generate equivalent atoms:

#1 -x+2,-y+1,-z+1

Table S4. Anisotropic displacement parameters ($Å^2 \times 10^3$) for **1,4-btmgbH$_2$(HCO$_3^-$)$_2$**. The anisotropic displacement factor exponent takes the form: $-2\pi^2[h^2 a^{*2}U^{11} + ... + 2 h k a^* b^* U^{12}]$

	U^{11}	U^{22}	U^{33}	U^{23}	U^{13}	U^{12}
O(1)	16(1)	22(1)	23(1)	-2(1)	2(1)	3(1)
O(2)	14(1)	26(1)	25(1)	-4(1)	-2(1)	7(1)
O(3)	17(1)	26(1)	17(1)	4(1)	2(1)	8(1)
C(9)	10(1)	15(1)	20(1)	5(1)	4(1)	0(1)
N(1)	10(1)	17(1)	12(1)	2(1)	0(1)	2(1)
N(2)	15(1)	17(1)	12(1)	-3(1)	2(1)	-2(1)

N(3)	16(1)	15(1)	11(1)	0(1)	-1(1)	3(1)
C(1)	10(1)	14(1)	12(1)	1(1)	-2(1)	0(1)
C(2)	11(1)	15(1)	13(1)	-1(1)	1(1)	2(1)
C(3)	13(1)	17(1)	10(1)	-1(1)	2(1)	0(1)
C(4)	13(1)	11(1)	12(1)	-1(1)	1(1)	3(1)
C(5)	19(1)	25(1)	17(1)	-7(1)	2(1)	-6(1)
C(6)	19(1)	27(1)	18(1)	-4(1)	7(1)	-5(1)
C(7)	18(1)	20(1)	17(1)	-5(1)	-4(1)	2(1)
C(8)	27(1)	21(1)	13(1)	4(1)	3(1)	7(1)

Table S5. Hydrogen coordinates (x 10^4) and isotropic displacement parameters (Å^2 x 10^3) for **1,4-btmgbH$_2$(HCO$_3^-$)$_2$**.

	x	y	z	U(eq)
H(2)	4203(18)	9980(20)	4315(13)	42(5)
H(1)	7710(18)	7100(20)	3456(12)	37(4)
H(2A)	8326(13)	6931(17)	5356(9)	11(3)
H(3)	10580(13)	4732(16)	3369(10)	11(3)
H(5A)	10234(16)	9185(18)	4190(11)	24(4)
H(5B)	11407(16)	10100(20)	3673(12)	33(4)
H(5C)	11690(16)	8330(20)	4178(12)	29(4)
H(6A)	12378(16)	7901(19)	2457(11)	27(4)
H(6B)	11185(14)	7471(18)	1561(11)	20(4)
H(6C)	11593(16)	9350(20)	1832(12)	32(4)
H(7A)	6479(16)	6906(18)	1571(11)	23(4)
H(7B)	7446(16)	5884(19)	892(12)	27(4)
H(7C)	7455(14)	5441(19)	2054(11)	22(4)
H(8A)	7800(16)	8920(19)	623(11)	26(4)
H(8B)	9288(16)	8152(19)	506(12)	26(4)
H(8C)	9149(15)	9659(19)	1250(11)	22(4)

Table S6. Torsion angles [°] for **1,4-btmgbH$_2$(HCO$_3^-$)$_2$**.

C(4)-N(1)-C(1)-C(2)	147.06(10)
C(4)-N(1)-C(1)-C(3)	-36.75(15)
C(3)-C(1)-C(2)-C(3)#1	0.13(17)
N(1)-C(1)-C(2)-C(3)#1	176.35(10)
C(2)-C(1)-C(3)-C(2)#1	-0.14(17)
N(1)-C(1)-C(3)-C(2)#1	-176.28(10)
C(8)-N(3)-C(4)-N(2)	-23.72(16)
C(7)-N(3)-C(4)-N(2)	161.23(10)
C(8)-N(3)-C(4)-N(1)	153.44(10)
C(7)-N(3)-C(4)-N(1)	-21.60(15)
C(5)-N(2)-C(4)-N(3)	151.54(11)
C(6)-N(2)-C(4)-N(3)	-33.49(16)
C(5)-N(2)-C(4)-N(1)	-25.58(16)
C(6)-N(2)-C(4)-N(1)	149.39(11)
C(1)-N(1)-C(4)-N(3)	137.97(10)
C(1)-N(1)-C(4)-N(2)	-44.81(15)

Symmetry transformations used to generate equivalent atoms:

#1 -x+2,-y+1,-z+1

Table S7. Hydrogen bonds for **1,4-btmgbH$_2$(HCO$_3^-$)$_2$** [Å and °].

D-H...A	d(D-H)	d(H...A)	d(D...A)	<(DHA)
N(1)-H(1)...O(1)	0.912(18)	1.807(18)	2.7135(13)	172.8(16)
O(2)-H(2)...O(3)#2	0.950(19)	1.671(19)	2.6145(13)	171.6(17)

Symmetry transformations used to generate equivalent atoms:

#1 -x+2,-y+1,-z+1 #2 -x+1,-y+2,-z+1

Table S8. Crystal data and structure refinement for **1,4-btmgbH$_2$(PF$_6$)$_2$**.

Empirical formula	[C_{16} H_{30} N_6] [F_6 P]$_2$	
Formula weight	596.40	
Temperature	133(2) K	
Wavelength	0.71073 Å	
Crystal system	Monoclinic	
Space group	$P2_1/c$	
Unit cell dimensions	a = 10.7063(5) Å	α = 90°.
	b = 8.9270(4) Å	β = 99.4282(8)°.
	c = 12.8565(6) Å	γ = 90°.
Volume	1212.16(10) Å^3	
Z	2	
Density (calculated)	1.634 Mg/m^3	
Absorption coefficient	0.290 mm^{-1}	
F(000)	612	
Crystal color	colorless	
Crystal size	0.305 x 0.283 x 0.124 mm^3	
Theta range for data collection	1.928 to 30.990°	
Index ranges	$-15 \leq h \leq 15$, $-12 \leq k \leq 12$, $-18 \leq l \leq 18$	
Reflections collected	29110	
Independent reflections	3703 [R(int) = 0.0338]	
Completeness to theta = 25.242°	100.0 %	
Absorption correction	Semi-empirical from equivalents	
Max. and min. transmission	0.8622 and 0.8117	
Refinement method	Full-matrix least-squares on F^2	
Data / restraints / parameters	3703 / 0 / 223	
Goodness-of-fit on F^2	1.025	
Final R indices [I>2sigma(I) = 3137 data]	R1 = 0.0311, wR2 = 0.0760	
R indices (all data, 0.69 Å)	R1 = 0.0396, wR2 = 0.0805	
Largest diff. peak and hole	0.426 and -0.336 e.Å^{-3}	

Table S9. Atomic coordinates (x 10^4) and equivalent isotropic displacement parameters ($Å^2$ x 10^3) for **1,4-btmgbH$_2$(PF$_6$)$_2$**. U(eq) is defined as one third of the trace of the orthogonalized U^{ij} tensor.

	x	y	z	U(eq)
P(1)	7564(1)	9078(1)	1768(1)	15(1)
F(1)	6450(1)	9386(1)	777(1)	26(1)
F(2)	8655(1)	8786(1)	2750(1)	35(1)
F(3)	7192(1)	10615(1)	2287(1)	24(1)
F(4)	7912(1)	7550(1)	1235(1)	28(1)
F(5)	6576(1)	8188(1)	2343(1)	26(1)
F(6)	8533(1)	9970(1)	1177(1)	31(1)
N(1)	3969(1)	7731(1)	536(1)	14(1)
N(2)	1793(1)	7262(1)	280(1)	16(1)
N(3)	2687(1)	9116(1)	1443(1)	15(1)
C(1)	4456(1)	6333(1)	258(1)	12(1)
C(2)	5452(1)	6350(1)	-314(1)	14(1)
C(3)	4003(1)	4977(1)	573(1)	14(1)
C(4)	2802(1)	8025(1)	751(1)	13(1)
C(5)	1720(1)	6630(1)	-777(1)	20(1)
C(6)	703(1)	6958(2)	798(1)	22(1)
C(7)	1574(1)	10092(2)	1344(1)	22(1)
C(8)	3773(1)	9609(1)	2207(1)	19(1)

Table S10. Bond lengths [Å] and angles [°] for **1,4-btmgbH$_2$(PF$_6$)$_2$**.

P(1)-F(2)	1.5945(8)
P(1)-F(6)	1.5950(8)
P(1)-F(4)	1.5968(8)
P(1)-F(5)	1.5987(8)
P(1)-F(3)	1.6048(8)
P(1)-F(1)	1.6197(8)
N(1)-C(4)	1.3489(14)
N(1)-C(1)	1.4208(13)

N(1)-H(1)	0.821(16)
N(2)-C(4)	1.3350(14)
N(2)-C(5)	1.4608(14)
N(2)-C(6)	1.4611(15)
N(3)-C(4)	1.3383(13)
N(3)-C(8)	1.4616(14)
N(3)-C(7)	1.4641(14)
C(1)-C(3)	1.3891(15)
C(1)-C(2)	1.3905(14)
C(2)-C(3)#1	1.3854(15)
C(2)-H(2)	0.928(16)
C(3)-C(2)#1	1.3854(15)
C(3)-H(3)	0.906(16)
C(5)-H(5A)	0.975(16)
C(5)-H(5B)	0.951(16)
C(5)-H(5C)	0.976(16)
C(6)-H(6A)	0.979(17)
C(6)-H(6B)	0.959(17)
C(6)-H(6C)	0.955(16)
C(7)-H(7A)	0.937(16)
C(7)-H(7B)	0.978(19)
C(7)-H(7C)	0.987(19)
C(8)-H(8A)	0.966(16)
C(8)-H(8B)	0.959(16)
C(8)-H(8C)	0.976(18)
F(2)-P(1)-F(6)	90.59(5)
F(2)-P(1)-F(4)	90.48(5)
F(6)-P(1)-F(4)	90.40(4)
F(2)-P(1)-F(5)	90.38(5)
F(6)-P(1)-F(5)	179.03(5)
F(4)-P(1)-F(5)	89.58(4)
F(2)-P(1)-F(3)	90.57(5)
F(6)-P(1)-F(3)	89.81(4)
F(4)-P(1)-F(3)	178.93(5)
F(5)-P(1)-F(3)	90.19(4)
F(2)-P(1)-F(1)	179.42(5)

F(6)-P(1)-F(1)	89.59(5)
F(4)-P(1)-F(1)	90.07(4)
F(5)-P(1)-F(1)	89.44(4)
F(3)-P(1)-F(1)	88.88(4)
C(4)-N(1)-C(1)	127.81(9)
C(4)-N(1)-H(1)	117.8(11)
C(1)-N(1)-H(1)	114.1(11)
C(4)-N(2)-C(5)	122.40(10)
C(4)-N(2)-C(6)	122.32(10)
C(5)-N(2)-C(6)	115.25(9)
C(4)-N(3)-C(8)	121.03(9)
C(4)-N(3)-C(7)	122.48(9)
C(8)-N(3)-C(7)	115.22(9)
C(3)-C(1)-C(2)	119.90(9)
C(3)-C(1)-N(1)	122.13(9)
C(2)-C(1)-N(1)	117.92(9)
C(3)#1-C(2)-C(1)	120.49(10)
C(3)#1-C(2)-H(2)	120.8(10)
C(1)-C(2)-H(2)	118.7(10)
C(2)#1-C(3)-C(1)	119.61(9)
C(2)#1-C(3)-H(3)	119.1(10)
C(1)-C(3)-H(3)	121.2(10)
N(2)-C(4)-N(3)	121.13(10)
N(2)-C(4)-N(1)	121.01(10)
N(3)-C(4)-N(1)	117.85(10)
N(2)-C(5)-H(5A)	110.6(9)
N(2)-C(5)-H(5B)	109.8(9)
H(5A)-C(5)-H(5B)	111.2(13)
N(2)-C(5)-H(5C)	107.7(10)
H(5A)-C(5)-H(5C)	108.2(13)
H(5B)-C(5)-H(5C)	109.3(13)
N(2)-C(6)-H(6A)	107.2(10)
N(2)-C(6)-H(6B)	111.6(10)
H(6A)-C(6)-H(6B)	109.6(14)
N(2)-C(6)-H(6C)	110.0(10)
H(6A)-C(6)-H(6C)	106.4(14)

H(6B)-C(6)-H(6C)	111.7(14)
N(3)-C(7)-H(7A)	109.9(10)
N(3)-C(7)-H(7B)	109.8(11)
H(7A)-C(7)-H(7B)	111.0(14)
N(3)-C(7)-H(7C)	109.0(11)
H(7A)-C(7)-H(7C)	107.5(14)
H(7B)-C(7)-H(7C)	109.5(15)
N(3)-C(8)-H(8A)	107.1(10)
N(3)-C(8)-H(8B)	109.3(9)
H(8A)-C(8)-H(8B)	111.6(13)
N(3)-C(8)-H(8C)	110.7(10)
H(8A)-C(8)-H(8C)	107.7(14)
H(8B)-C(8)-H(8C)	110.4(14)

Symmetry transformations used to generate equivalent atoms:

#1 -x+1,-y+1,-z

Table S11. Anisotropic displacement parameters (Å^2 x 10^3) for **1,4-btmgbH$_2$(PF$_6$)$_2$**. The anisotropic displacement factor exponent takes the form: $-2\pi^2[h^2 a^{*2}U^{11} + ... + 2 h k a^* b^* U^{12}]$

	U^{11}	U^{22}	U^{33}	U^{23}	U^{13}	U^{12}
P(1)	14(1)	14(1)	19(1)	0(1)	5(1)	-1(1)
F(1)	27(1)	23(1)	24(1)	1(1)	-4(1)	-1(1)
F(2)	31(1)	31(1)	37(1)	6(1)	-14(1)	-3(1)
F(3)	34(1)	16(1)	24(1)	-3(1)	11(1)	-2(1)
F(4)	27(1)	21(1)	40(1)	-6(1)	13(1)	4(1)
F(5)	26(1)	19(1)	36(1)	5(1)	16(1)	-2(1)
F(6)	25(1)	28(1)	44(1)	4(1)	19(1)	-5(1)
N(1)	13(1)	11(1)	20(1)	-3(1)	5(1)	0(1)
N(2)	14(1)	16(1)	17(1)	-2(1)	2(1)	1(1)
N(3)	13(1)	14(1)	16(1)	-2(1)	2(1)	4(1)
C(1)	13(1)	12(1)	12(1)	-1(1)	1(1)	2(1)
C(2)	15(1)	12(1)	15(1)	1(1)	4(1)	0(1)
C(3)	13(1)	15(1)	14(1)	1(1)	5(1)	1(1)

C(4)	14(1)	11(1)	14(1)	1(1)	2(1)	3(1)
C(5)	20(1)	20(1)	18(1)	-4(1)	-1(1)	1(1)
C(6)	14(1)	26(1)	27(1)	-1(1)	5(1)	-1(1)
C(7)	21(1)	22(1)	24(1)	-5(1)	1(1)	11(1)
C(8)	18(1)	20(1)	20(1)	-6(1)	1(1)	1(1)

Table S12. Hydrogen coordinates (x 10^4) and isotropic displacement parameters (Å^2 x 10^3) for **1,4-btmgbH$_2$(PF$_6$)$_2$**.

	x	y	z	U(eq)
H(1)	4497(15)	8406(18)	630(12)	21(4)
H(2)	5746(14)	7267(18)	-514(12)	21(4)
H(3)	3357(15)	4942(17)	948(12)	20(4)
H(5A)	1798(15)	5542(18)	-742(12)	21(4)
H(5B)	2357(15)	7061(18)	-1118(12)	21(4)
H(5C)	888(16)	6876(19)	-1173(12)	27(4)
H(6A)	567(16)	5870(20)	775(13)	29(4)
H(6B)	-44(16)	7460(20)	450(13)	31(4)
H(6C)	894(15)	7225(19)	1526(13)	25(4)
H(7A)	1034(15)	9893(18)	708(12)	22(4)
H(7B)	1137(17)	9950(20)	1947(14)	38(5)
H(7C)	1851(17)	11140(20)	1322(14)	38(5)
H(8A)	3445(15)	10047(18)	2793(13)	24(4)
H(8B)	4308(14)	8766(18)	2423(12)	20(4)
H(8C)	4246(16)	10380(20)	1903(13)	30(4)

Table S13. Torsion angles [°] for **1,4-btmgbH$_2$(PF$_6$)$_2$**.

C(4)-N(1)-C(1)-C(3)	-27.09(16)
C(4)-N(1)-C(1)-C(2)	155.48(10)
C(3)-C(1)-C(2)-C(3)#1	0.03(17)
N(1)-C(1)-C(2)-C(3)#1	177.51(10)
C(2)-C(1)-C(3)-C(2)#1	-0.03(17)
N(1)-C(1)-C(3)-C(2)#1	-177.41(10)
C(5)-N(2)-C(4)-N(3)	149.95(10)
C(6)-N(2)-C(4)-N(3)	-32.19(16)
C(5)-N(2)-C(4)-N(1)	-29.32(16)
C(6)-N(2)-C(4)-N(1)	148.54(11)
C(8)-N(3)-C(4)-N(2)	159.63(11)
C(7)-N(3)-C(4)-N(2)	-33.85(16)
C(8)-N(3)-C(4)-N(1)	-21.07(15)
C(7)-N(3)-C(4)-N(1)	145.44(11)
C(1)-N(1)-C(4)-N(2)	-31.86(16)
C(1)-N(1)-C(4)-N(3)	148.85(10)

Symmetry transformations used to generate equivalent atoms:

#1 -x+1,-y+1,-z

Table S14. Crystal data and structure refinement for **1,4-btmgbH[PF$_6$]**.

Empirical formula	[C_{16} H_{29} N_6] [PF_6]	
Formula weight	450.42	
Temperature	93(2) K	
Wavelength	1.54178 Å	
Crystal system	Monoclinic	
Space group	$P2_1/c$	
Unit cell dimensions	a = 13.5006(10) Å	α = 90°.
	b = 11.7654(9) Å	β = 116.210(4)°.
	c = 14.5989(11) Å	γ = 90°.
Volume	2080.5(3) Å^3	
Z	4	
Density (calculated)	1.438 Mg/m^3	
Absorption coefficient	1.800 mm^{-1}	
F(000)	944	
Crystal color	colorless	
Crystal size	0.154 x 0.127 x 0.118 mm^3	
Theta range for data collection	3.649 to 69.128°	
Index ranges	$-15 \leq h \leq 16$, $-14 \leq k \leq 14$, $-17 \leq l \leq 17$	
Reflections collected	35044	
Independent reflections	3814 [R(int) = 0.0617]	
Completeness to theta = 67.679°	98.7 %	
Absorption correction	Semi-empirical from equivalents	
Max. and min. transmission	0.7532 and 0.6100	
Refinement method	Full-matrix least-squares on F^2	
Data / restraints / parameters	3814 / 0 / 274	
Goodness-of-fit on F^2	1.027	
Final R indices [I>2sigma(I) = 3054 data]	R1 = 0.0386, wR2 = 0.1037	
R indices (all data, 0.825 Å)	R1 = 0.0504, wR2 = 0.1132	
Largest diff. peak and hole	0.258 and -0.381 e.Å^{-3}	

Table S15. Atomic coordinates (x 10^4) and equivalent isotropic displacement parameters (Å^2 x 10^3)

for **1,4-btmgbH[PF$_6$]**. U(eq) is defined as one third of the trace of the orthogonalized U^{ij} tensor.

	x	y	z	U(eq)
P(1)	9952(1)	9551(1)	7433(1)	21(1)
F(1)	10034(1)	8202(1)	7466(1)	26(1)
F(2)	8633(1)	9460(1)	6831(1)	37(1)
F(3)	9867(1)	10898(1)	7400(1)	40(1)
F(4)	11271(1)	9633(1)	8033(1)	36(1)
F(5)	10036(1)	9560(1)	6368(1)	31(1)
F(6)	9873(1)	9545(1)	8500(1)	32(1)
N(1)	5904(1)	7591(1)	3559(1)	17(1)
N(2)	7343(1)	8188(1)	3222(1)	19(1)

N(3)	7679(1)	6875(1)	4518(1)	19(1)
N(4)	4309(1)	7267(1)	6529(1)	17(1)
N(5)	2836(1)	6768(1)	6851(1)	19(1)
N(6)	2526(1)	8027(1)	5530(1)	19(1)
C(1)	5513(1)	7508(2)	4317(1)	16(1)
C(2)	6041(1)	8061(1)	5246(1)	17(1)
C(3)	5625(1)	7990(1)	5961(1)	17(1)
C(4)	4662(1)	7376(1)	5758(1)	16(1)
C(5)	4133(1)	6829(1)	4813(1)	18(1)
C(6)	4555(1)	6890(1)	4102(1)	18(1)
C(7)	6983(1)	7553(1)	3777(1)	16(1)
C(8)	6784(1)	9223(2)	2698(1)	23(1)
C(9)	8266(1)	7835(2)	3013(1)	25(1)
C(10)	8840(1)	7166(2)	5150(1)	27(1)
C(11)	7312(2)	5844(2)	4832(1)	23(1)
C(12)	3244(1)	7350(1)	6288(1)	17(1)
C(13)	3394(1)	5772(2)	7438(1)	22(1)
C(14)	1942(1)	7204(2)	7060(1)	25(1)
C(15)	2885(2)	9033(2)	5185(1)	24(1)
C(16)	1380(1)	7702(2)	4883(1)	28(1)

Table S16. Bond lengths [Å] and angles [°] for **1,4-btmgbH[PF_6]**.

P(1)-F(3)	1.5886(12)
P(1)-F(1)	1.5893(11)
P(1)-F(4)	1.6028(11)
P(1)-F(2)	1.6045(11)
P(1)-F(5)	1.6072(11)
P(1)-F(6)	1.6075(11)
N(1)-C(7)	1.347(2)
N(1)-C(1)	1.425(2)
N(1)-H(1)	0.91(2)
N(2)-C(7)	1.341(2)
N(2)-C(8)	1.459(2)

N(2)-C(9)	1.467(2)
N(3)-C(7)	1.339(2)
N(3)-C(11)	1.459(2)
N(3)-C(10)	1.465(2)
N(4)-C(12)	1.323(2)
N(4)-C(4)	1.409(2)
N(5)-C(12)	1.360(2)
N(5)-C(13)	1.451(2)
N(5)-C(14)	1.462(2)
N(6)-C(12)	1.360(2)
N(6)-C(15)	1.452(2)
N(6)-C(16)	1.463(2)
C(1)-C(2)	1.384(2)
C(1)-C(6)	1.394(2)
C(2)-C(3)	1.389(2)
C(2)-H(2)	0.9500
C(3)-C(4)	1.400(2)
C(3)-H(3)	0.9500
C(4)-C(5)	1.399(2)
C(5)-C(6)	1.389(2)
C(5)-H(5)	0.9500
C(6)-H(6)	0.9500
C(8)-H(8A)	0.9800
C(8)-H(8B)	0.9800
C(8)-H(8C)	0.9800
C(9)-H(9A)	0.9800
C(9)-H(9B)	0.9800
C(9)-H(9C)	0.9800
C(10)-H(10A)	0.9800
C(10)-H(10B)	0.9800
C(10)-H(10C)	0.9800
C(11)-H(11A)	0.9800
C(11)-H(11B)	0.9800
C(11)-H(11C)	0.9800
C(13)-H(12A)	0.9800
C(13)-H(12B)	0.9800

C(13)-H(12C)	0.9800
C(14)-H(13A)	0.9800
C(14)-H(13B)	0.9800
C(14)-H(13C)	0.9800
C(15)-H(15A)	0.9800
C(15)-H(15B)	0.9800
C(15)-H(15C)	0.9800
C(16)-H(14A)	0.9800
C(16)-H(14B)	0.9800
C(16)-H(14C)	0.9800
F(3)-P(1)-F(1)	179.82(6)
F(3)-P(1)-F(4)	90.26(7)
F(1)-P(1)-F(4)	89.91(6)
F(3)-P(1)-F(2)	90.09(7)
F(1)-P(1)-F(2)	89.75(6)
F(4)-P(1)-F(2)	179.65(8)
F(3)-P(1)-F(5)	89.90(6)
F(1)-P(1)-F(5)	90.18(5)
F(4)-P(1)-F(5)	89.59(6)
F(2)-P(1)-F(5)	90.33(6)
F(3)-P(1)-F(6)	89.96(6)
F(1)-P(1)-F(6)	89.97(6)
F(4)-P(1)-F(6)	90.22(6)
F(2)-P(1)-F(6)	89.87(6)
F(5)-P(1)-F(6)	179.76(7)
C(7)-N(1)-C(1)	123.19(14)
C(7)-N(1)-H(1)	117.7(14)
C(1)-N(1)-H(1)	119.1(14)
C(7)-N(2)-C(8)	122.71(14)
C(7)-N(2)-C(9)	122.37(15)
C(8)-N(2)-C(9)	114.67(13)
C(7)-N(3)-C(11)	122.32(14)
C(7)-N(3)-C(10)	122.95(15)
C(11)-N(3)-C(10)	114.42(14)
C(12)-N(4)-C(4)	119.40(13)
C(12)-N(5)-C(13)	121.41(14)

C(12)-N(5)-C(14)	122.83(15)
C(13)-N(5)-C(14)	114.86(13)
C(12)-N(6)-C(15)	122.12(14)
C(12)-N(6)-C(16)	122.87(15)
C(15)-N(6)-C(16)	114.18(14)
C(2)-C(1)-C(6)	119.37(14)
C(2)-C(1)-N(1)	121.47(14)
C(6)-C(1)-N(1)	119.11(14)
C(1)-C(2)-C(3)	120.24(15)
C(1)-C(2)-H(2)	119.9
C(3)-C(2)-H(2)	119.9
C(2)-C(3)-C(4)	121.21(15)
C(2)-C(3)-H(3)	119.4
C(4)-C(3)-H(3)	119.4
C(5)-C(4)-C(3)	117.91(14)
C(5)-C(4)-N(4)	122.71(14)
C(3)-C(4)-N(4)	119.23(14)
C(6)-C(5)-C(4)	120.91(15)
C(6)-C(5)-H(5)	119.5
C(4)-C(5)-H(5)	119.5
C(5)-C(6)-C(1)	120.35(15)
C(5)-C(6)-H(6)	119.8
C(1)-C(6)-H(6)	119.8
N(3)-C(7)-N(2)	120.53(14)
N(3)-C(7)-N(1)	120.69(15)
N(2)-C(7)-N(1)	118.77(15)
N(2)-C(8)-H(8A)	109.5
N(2)-C(8)-H(8B)	109.5
H(8A)-C(8)-H(8B)	109.5
N(2)-C(8)-H(8C)	109.5
H(8A)-C(8)-H(8C)	109.5
H(8B)-C(8)-H(8C)	109.5
N(2)-C(9)-H(9A)	109.5
N(2)-C(9)-H(9B)	109.5
H(9A)-C(9)-H(9B)	109.5
N(2)-C(9)-H(9C)	109.5

H(9A)-C(9)-H(9C)	109.5
H(9B)-C(9)-H(9C)	109.5
N(3)-C(10)-H(10A)	109.5
N(3)-C(10)-H(10B)	109.5
H(10A)-C(10)-H(10B)	109.5
N(3)-C(10)-H(10C)	109.5
H(10A)-C(10)-H(10C)	109.5
H(10B)-C(10)-H(10C)	109.5
N(3)-C(11)-H(11A)	109.5
N(3)-C(11)-H(11B)	109.5
H(11A)-C(11)-H(11B)	109.5
N(3)-C(11)-H(11C)	109.5
H(11A)-C(11)-H(11C)	109.5
H(11B)-C(11)-H(11C)	109.5
N(4)-C(12)-N(5)	118.93(15)
N(4)-C(12)-N(6)	124.10(15)
N(5)-C(12)-N(6)	116.89(14)
N(5)-C(13)-H(12A)	109.5
N(5)-C(13)-H(12B)	109.5
H(12A)-C(13)-H(12B)	109.5
N(5)-C(13)-H(12C)	109.5
H(12A)-C(13)-H(12C)	109.5
H(12B)-C(13)-H(12C)	109.5
N(5)-C(14)-H(13A)	109.5
N(5)-C(14)-H(13B)	109.5
H(13A)-C(14)-H(13B)	109.5
N(5)-C(14)-H(13C)	109.5
H(13A)-C(14)-H(13C)	109.5
H(13B)-C(14)-H(13C)	109.5
N(6)-C(15)-H(15A)	109.5
N(6)-C(15)-H(15B)	109.5
H(15A)-C(15)-H(15B)	109.5
N(6)-C(15)-H(15C)	109.5
H(15A)-C(15)-H(15C)	109.5
H(15B)-C(15)-H(15C)	109.5
N(6)-C(16)-H(14A)	109.5

N(6)-C(16)-H(14B)	109.5
H(14A)-C(16)-H(14B)	109.5
N(6)-C(16)-H(14C)	109.5
H(14A)-C(16)-H(14C)	109.5
H(14B)-C(16)-H(14C)	109.5

Table S17. Anisotropic displacement parameters ($Å^2 \times 10^3$) for **1,4-btmgbH[PF$_6$]**. The anisotropic displacement factor exponent takes the form: $-2\pi^2[h^2 a^{*2}U^{11} + ... + 2 h k a^* b^* U^{12}]$.

	U^{11}	U^{22}	U^{33}	U^{23}	U^{13}	U^{12}
P(1)	24(1)	20(1)	23(1)	0(1)	15(1)	-1(1)
F(1)	32(1)	21(1)	25(1)	1(1)	13(1)	0(1)
F(2)	24(1)	44(1)	40(1)	16(1)	12(1)	7(1)
F(3)	65(1)	21(1)	59(1)	2(1)	50(1)	1(1)
F(4)	28(1)	45(1)	37(1)	-11(1)	16(1)	-12(1)
F(5)	47(1)	30(1)	26(1)	1(1)	24(1)	-1(1)
sF(6)	45(1)	31(1)	30(1)	-3(1)	26(1)	-4(1)
N(1)	13(1)	30(1)	12(1)	0(1)	8(1)	-1(1)
N(2)	16(1)	26(1)	19(1)	-1(1)	12(1)	-1(1)
N(3)	14(1)	28(1)	14(1)	0(1)	6(1)	0(1)
N(4)	13(1)	29(1)	12(1)	0(1)	8(1)	-1(1)
N(5)	15(1)	28(1)	19(1)	2(1)	12(1)	0(1)
N(6)	13(1)	29(1)	15(1)	2(1)	7(1)	0(1)
C(1)	13(1)	25(1)	13(1)	2(1)	9(1)	2(1)
C(2)	12(1)	24(1)	15(1)	2(1)	7(1)	-2(1)
C(3)	15(1)	24(1)	12(1)	-2(1)	6(1)	-1(1)
C(4)	14(1)	24(1)	12(1)	3(1)	7(1)	2(1)
C(5)	13(1)	25(1)	16(1)	1(1)	7(1)	-3(1)
C(6)	15(1)	25(1)	13(1)	-2(1)	7(1)	-2(1)
C(7)	15(1)	24(1)	13(1)	-4(1)	9(1)	-2(1)
C(8)	25(1)	24(1)	23(1)	0(1)	14(1)	-2(1)
C(9)	19(1)	37(1)	25(1)	0(1)	16(1)	0(1)
C(10)	15(1)	44(1)	19(1)	-2(1)	3(1)	0(1)

C(11)	25(1)	26(1)	19(1)	2(1)	11(1)	3(1)
C(12)	16(1)	23(1)	13(1)	-4(1)	7(1)	-3(1)
C(13)	24(1)	26(1)	21(1)	1(1)	13(1)	-3(1)
C(14)	19(1)	39(1)	23(1)	0(1)	15(1)	0(1)
C(15)	27(1)	28(1)	21(1)	2(1)	14(1)	3(1)
C(16)	16(1)	44(1)	20(1)	0(1)	3(1)	-1(1)

Table S18. Hydrogen coordinates (x 10^4) and isotropic displacement parameters (Å^2 x 10^3) for **1,4-btmgbH[PF$_6$]**.

	x	y	z	U(eq)
H(1)	5410(20)	7710(20)	2895(19)	45(7)
H(2)	6691	8490	5396	20
H(3)	6000	8366	6599	20
H(5)	3476	6409	4656	21
H(6)	4188	6509	3466	21
H(8A)	6404	9083	1961	34
H(8B)	7328	9831	2839	34
H(8C)	6243	9451	2942	34
H(9A)	8900	8334	3382	37
H(9B)	8045	7888	2279	37
H(9C)	8469	7049	3240	37
H(10A)	9312	6652	4989	41
H(10B)	9009	7088	5873	41
H(10C)	8973	7951	5012	41
H(11A)	7297	5976	5489	35
H(11B)	7823	5221	4903	35
H(11C)	6570	5643	4317	35
H(12A)	3938	5503	7214	33
H(12B)	2851	5171	7332	33
H(12C)	3768	5968	8165	33
H(13A)	1732	7965	6765	38

H(13B)	2192	7244	7800	38
H(13C)	1304	6695	6757	38
H(15A)	3630	9243	5690	36
H(15B)	2376	9661	5102	36
H(15C)	2893	8874	4529	36
H(14A)	1241	6944	5080	42
H(14B)	1249	7691	4167	42
H(14C)	882	8252	4969	42

Table S19. Torsion angles [°] for **1,4-btmgbH[PF_6]**.

C(7)-N(1)-C(1)-C(2)	41.2(2)
C(7)-N(1)-C(1)-C(6)	-141.08(17)
C(6)-C(1)-C(2)-C(3)	0.6(2)
N(1)-C(1)-C(2)-C(3)	178.30(15)
C(1)-C(2)-C(3)-C(4)	-0.8(3)
C(2)-C(3)-C(4)-C(5)	0.3(2)
C(2)-C(3)-C(4)-N(4)	175.98(15)
C(12)-N(4)-C(4)-C(5)	-45.3(2)
C(12)-N(4)-C(4)-C(3)	139.29(16)
C(3)-C(4)-C(5)-C(6)	0.3(2)
N(4)-C(4)-C(5)-C(6)	-175.20(15)
C(4)-C(5)-C(6)-C(1)	-0.4(3)
C(2)-C(1)-C(6)-C(5)	0.0(3)
N(1)-C(1)-C(6)-C(5)	-177.74(15)
C(11)-N(3)-C(7)-N(2)	-152.52(15)
C(10)-N(3)-C(7)-N(2)	34.3(2)
C(11)-N(3)-C(7)-N(1)	25.9(2)
C(10)-N(3)-C(7)-N(1)	-147.27(16)
C(8)-N(2)-C(7)-N(3)	-156.10(15)
C(9)-N(2)-C(7)-N(3)	29.9(2)
C(8)-N(2)-C(7)-N(1)	25.4(2)
C(9)-N(2)-C(7)-N(1)	-148.55(15)
C(1)-N(1)-C(7)-N(3)	36.4(2)

C(1)-N(1)-C(7)-N(2)	-145.06(16)
C(4)-N(4)-C(12)-N(5)	150.27(15)
C(4)-N(4)-C(12)-N(6)	-32.8(2)
C(13)-N(5)-C(12)-N(4)	-22.9(2)
C(14)-N(5)-C(12)-N(4)	145.63(16)
C(13)-N(5)-C(12)-N(6)	159.92(15)
C(14)-N(5)-C(12)-N(6)	-31.5(2)
C(15)-N(6)-C(12)-N(4)	-24.9(2)
C(16)-N(6)-C(12)-N(4)	143.99(17)
C(15)-N(6)-C(12)-N(5)	152.04(15)
C(16)-N(6)-C(12)-N(5)	-39.0(2)

Table S20. Crystal data and structure refinement for **1,4-btmgbH$_2$(HCO$_3^-$)$_2$•(H$_2$O)$_2$.**

Empirical formula	$[C_{16} H_{30} N_6] [C H O_3]_2 (H_2 O)_2$	
Formula weight	464.53	
Temperature	92(2) K	
Wavelength	1.54178 Å	
Crystal system	Triclinic	
Space group	*P*-1	
Unit cell dimensions	a = 7.9540(9) Å	α = 87.747(6)°.
	b = 9.1652(11) Å	β = 66.175(5)°.
	c = 9.2032(11) Å	γ = 69.028(5)°.
Volume	568.71(12) Å^3	
Z	1	
Density (calculated)	1.356 Mg/m^3	
Absorption coefficient	0.899 mm^{-1}	
F(000)	250	
Crystal color	colorless	
Crystal size	0.192 x 0.139 x 0.097 mm^3	
Theta range for data collection	5.208 to 68.832°	
Index ranges	$-9 \leq h \leq 9$, $-11 \leq k \leq 11$, $-11 \leq l \leq 11$	
Reflections collected	28347	
Independent reflections	2089 [R(int) = 0.0475]	
Completeness to theta = 67.679°	99.6 %	
Absorption correction	Semi-empirical from equivalents	
Max. and min. transmission	0.7532 and 0.6591	
Refinement method	Full-matrix least-squares on F^2	
Data / restraints / parameters	2089 / 0 / 218	
Goodness-of-fit on F^2	1.094	
Final R indices [I>2sigma(I) = 1871 data]	R1 = 0.0321, wR2 = 0.0847	
R indices (all data, 0.83 Å)	R1 = 0.0358, wR2 = 0.0874	
Extinction coefficient	0.0049(10)	
Largest diff. peak and hole	0.244 and -0.202 e.Å^{-3}	

Table S21. Atomic coordinates (x 10^4) and equivalent isotropic displacement parameters (Å^2 x 10^3) for **1,4-btmgbH$_2$(HCO$_3^-$)$_2$•(H$_2$O)$_2$**. U(eq) is defined as one third of the trace of the orthogonalized U^{ij} tensor.

	x	y	z	U(eq)
O(1)	3692(1)	7941(1)	3230(1)	27(1)
O(2)	6194(1)	8382(1)	3329(1)	24(1)
O(3)	3143(2)	9618(1)	5214(1)	39(1)
C(9)	4258(2)	8652(2)	3962(2)	22(1)
O(4)	-518(2)	9592(1)	7213(1)	35(1)
N(1)	3695(2)	5008(1)	2803(1)	17(1)
N(2)	4958(2)	2665(1)	1119(1)	17(1)
N(3)	7076(2)	3585(1)	1602(1)	19(1)
C(1)	1830(2)	4972(1)	3886(1)	15(1)
C(2)	1636(2)	3595(2)	4489(2)	17(1)
C(3)	184(2)	6379(2)	4406(2)	17(1)
C(4)	5242(2)	3742(1)	1840(1)	16(1)
C(5)	7470(2)	4298(2)	2766(2)	24(1)
C(6)	8828(2)	2726(2)	144(2)	24(1)
C(7)	6138(2)	981(2)	935(2)	24(1)
C(8)	3251(2)	3040(2)	734(2)	21(1)

Table S22. Bond lengths [Å] and angles [°] for **1,4-btmgbH$_2$(HCO$_3^-$)$_2$•(H$_2$O)$_2$**.

O(1)-C(9)	1.2530(16)
O(2)-C(9)	1.3361(17)
O(2)-H(2)	0.91(2)
O(3)-C(9)	1.2610(17)
O(4)-H(4A)	0.90(2)
O(4)-H(4B)	0.88(2)
N(1)-C(4)	1.3503(16)
N(1)-C(1)	1.4259(16)
N(1)-H(1)	0.872(19)

N(2)-C(4)	1.3408(17)
N(2)-C(8)	1.4608(17)
N(2)-C(7)	1.4652(17)
N(3)-C(4)	1.3373(16)
N(3)-C(5)	1.4651(17)
N(3)-C(6)	1.4662(17)
C(1)-C(3)	1.3925(17)
C(1)-C(2)	1.3929(19)
C(2)-C(3)#1	1.3848(18)
C(2)-H(2A)	0.975(16)
C(3)-C(2)#1	1.3848(18)
C(3)-H(3)	0.975(16)
C(5)-H(5A)	0.983(18)
C(5)-H(5B)	0.979(18)
C(5)-H(5C)	0.97(2)
C(6)-H(6A)	1.004(17)
C(6)-H(6B)	0.992(17)
C(6)-H(6C)	0.983(18)
C(7)-H(7A)	0.976(17)
C(7)-H(7B)	0.993(17)
C(7)-H(7C)	0.986(17)
C(8)-H(8A)	0.975(17)
C(8)-H(8B)	0.994(17)
C(8)-H(8C)	0.983(17)
C(9)-O(2)-H(2)	108.9(13)
O(1)-C(9)-O(3)	125.19(13)
O(1)-C(9)-O(2)	116.58(12)
O(3)-C(9)-O(2)	118.21(12)
H(4A)-O(4)-H(4B)	107.8(19)
C(4)-N(1)-C(1)	124.38(11)
C(4)-N(1)-H(1)	118.7(11)
C(1)-N(1)-H(1)	116.1(11)
C(4)-N(2)-C(8)	122.63(11)
C(4)-N(2)-C(7)	121.56(11)
C(8)-N(2)-C(7)	114.90(11)
C(4)-N(3)-C(5)	122.23(11)

C(4)-N(3)-C(6)	122.65(11)
C(5)-N(3)-C(6)	115.10(11)
C(3)-C(1)-C(2)	119.59(12)
C(3)-C(1)-N(1)	118.40(11)
C(2)-C(1)-N(1)	121.93(11)
C(3)#1-C(2)-C(1)	120.29(12)
C(3)#1-C(2)-H(2A)	119.8(9)
C(1)-C(2)-H(2A)	119.9(9)
C(2)#1-C(3)-C(1)	120.12(12)
C(2)#1-C(3)-H(3)	120.1(9)
C(1)-C(3)-H(3)	119.7(9)
N(3)-C(4)-N(2)	120.26(11)
N(3)-C(4)-N(1)	118.84(11)
N(2)-C(4)-N(1)	120.90(11)
N(3)-C(5)-H(5A)	107.6(10)
N(3)-C(5)-H(5B)	109.3(10)
H(5A)-C(5)-H(5B)	109.9(14)
N(3)-C(5)-H(5C)	111.4(11)
H(5A)-C(5)-H(5C)	109.4(14)
H(5B)-C(5)-H(5C)	109.2(14)
N(3)-C(6)-H(6A)	108.4(9)
N(3)-C(6)-H(6B)	110.5(9)
H(6A)-C(6)-H(6B)	108.2(13)
N(3)-C(6)-H(6C)	109.8(9)
H(6A)-C(6)-H(6C)	109.9(13)
H(6B)-C(6)-H(6C)	110.0(13)
N(2)-C(7)-H(7A)	108.9(10)
N(2)-C(7)-H(7B)	107.2(9)
H(7A)-C(7)-H(7B)	110.5(14)
N(2)-C(7)-H(7C)	111.5(9)
H(7A)-C(7)-H(7C)	108.9(14)
H(7B)-C(7)-H(7C)	109.9(13)
N(2)-C(8)-H(8A)	109.3(9)
N(2)-C(8)-H(8B)	108.2(9)
H(8A)-C(8)-H(8B)	110.6(13)
N(2)-C(8)-H(8C)	111.1(9)

H(8A)-C(8)-H(8C)	109.6(13)
H(8B)-C(8)-H(8C)	108.0(13)

Symmetry transformations used to generate equivalent atoms:

#1 -x,-y+1,-z+1

Table S23. Anisotropic displacement parameters ($Å^2 \times 10^3$) for **1,4-btmgbH$_2$(HCO$_3^-$)$_2$●(H$_2$O)$_2$**. The anisotropic displacement factor exponent takes the form: $-2\pi^2[h^2 a^{*2}U^{11} + ... + 2 h k a^* b^* U^{12}]$

	U^{11}	U^{22}	U^{33}	U^{23}	U^{13}	U^{12}
O(1)	30(1)	21(1)	32(1)	-2(1)	-13(1)	-11(1)
O(2)	22(1)	23(1)	25(1)	-2(1)	-9(1)	-6(1)
O(3)	27(1)	49(1)	35(1)	-19(1)	1(1)	-20(1)
C(9)	24(1)	17(1)	24(1)	2(1)	-9(1)	-9(1)
O(4)	26(1)	40(1)	28(1)	8(1)	-5(1)	-9(1)
N(1)	14(1)	14(1)	18(1)	-2(1)	-3(1)	-4(1)
N(2)	16(1)	15(1)	16(1)	-1(1)	-5(1)	-3(1)
N(3)	13(1)	20(1)	18(1)	-3(1)	-4(1)	-3(1)
C(1)	13(1)	17(1)	13(1)	-2(1)	-4(1)	-4(1)
C(2)	15(1)	15(1)	17(1)	-2(1)	-6(1)	-1(1)
C(3)	18(1)	15(1)	17(1)	0(1)	-6(1)	-5(1)
C(4)	15(1)	16(1)	13(1)	2(1)	-4(1)	-4(1)
C(5)	18(1)	30(1)	24(1)	-4(1)	-9(1)	-6(1)
C(6)	15(1)	26(1)	21(1)	-3(1)	-2(1)	-3(1)
C(7)	25(1)	15(1)	25(1)	-2(1)	-9(1)	-3(1)
C(8)	20(1)	23(1)	19(1)	-1(1)	-8(1)	-7(1)

Table S24. Hydrogen coordinates (x 10^4) and isotropic displacement parameters (Å^2 x 10^3) for **1,4-btmgbH$_2$(HCO$_3^-$)$_2$●(H$_2$O)$_2$**.

	x	y	z	U(eq)
H(2)	6400(30)	9070(30)	3860(30)	52(6)
H(4A)	690(30)	9520(20)	6490(30)	49(6)
H(4B)	-1410(30)	10300(30)	6950(20)	53(6)
H(1)	3890(20)	5870(20)	2900(20)	28(4)
H(2A)	2790(20)	2611(19)	4143(18)	24(4)
H(3)	330(20)	7351(19)	4017(18)	23(4)
H(5A)	8640(30)	3520(20)	2860(20)	31(4)
H(5B)	6320(30)	4568(19)	3800(20)	33(4)
H(5C)	7730(30)	5240(20)	2420(20)	39(5)
H(6A)	9650(20)	3392(19)	-260(20)	29(4)
H(6B)	9650(20)	1730(20)	380(19)	27(4)
H(6C)	8420(20)	2508(19)	-670(20)	27(4)
H(7A)	6950(20)	823(19)	1530(20)	29(4)
H(7B)	5190(20)	436(19)	1380(20)	29(4)
H(7C)	7010(20)	563(19)	-200(20)	29(4)
H(8A)	2580(20)	4180(20)	863(19)	26(4)
H(8B)	3740(20)	2588(19)	-390(20)	27(4)
H(8C)	2320(20)	2569(19)	1428(19)	27(4)

Table S25. Torsion angles [°] for **1,4-btmgbH$_2$(HCO$_3^-$)$_2$•(H$_2$O)$_2$**.

C(4)-N(1)-C(1)-C(3)	-159.04(12)
C(4)-N(1)-C(1)-C(2)	24.16(18)
C(3)-C(1)-C(2)-C(3)#1	0.3(2)
N(1)-C(1)-C(2)-C(3)#1	177.05(11)
C(2)-C(1)-C(3)-C(2)#1	-0.3(2)
N(1)-C(1)-C(3)-C(2)#1	-177.17(11)
C(5)-N(3)-C(4)-N(2)	-155.21(12)
C(6)-N(3)-C(4)-N(2)	26.48(18)
C(5)-N(3)-C(4)-N(1)	25.42(18)
C(6)-N(3)-C(4)-N(1)	-152.88(12)
C(8)-N(2)-C(4)-N(3)	-152.58(12)
C(7)-N(2)-C(4)-N(3)	38.92(17)
C(8)-N(2)-C(4)-N(1)	26.77(18)
C(7)-N(2)-C(4)-N(1)	-141.73(12)
C(1)-N(1)-C(4)-N(3)	-141.95(12)
C(1)-N(1)-C(4)-N(2)	38.69(18)

Symmetry transformations used to generate equivalent atoms:

#1 -x,-y+1,-z+1

Table S26. Hydrogen bonds for **1,4-btmgbH$_2$(HCO$_3^-$)$_2$•(H$_2$O)$_2$** [Å and °].

D-H...A	d(D-H)	d(H...A)	d(D...A)	<(DHA)
N(1)-H(1)...O(1)	0.872(19)	1.875(19)	2.7313(15)	167.0(16)
O(4)-H(4A)...O(3)	0.90(2)	1.86(2)	2.7528(16)	171.0(19)

Symmetry transformations used to generate equivalent atoms:

#1 -x,-y+1,-z+1
